# fNIRS brain measures of ongoing nociception during surgical incisions under anesthesia

**DOI:** 10.1117/1.NPh.9.1.015002

**Published:** 2022-01-27

**Authors:** Stephen Green, Keerthana Deepti Karunakaran, Robert Labadie, Barry Kussman, Arielle Mizrahi-Arnaud, Andrea Gomez Morad, Delany Berry, David Zurakowski, Lyle Micheli, Ke Peng, David Borsook

**Affiliations:** a Boston Children’s Hospital, Harvard Medical School, The Center for Pain and the Brain, Department of Anesthesiology, Critical Care and Pain Medicine, Boston, Massachusetts, United States; b Boston Children’s Hospital, Harvard Medical School, Division of Cardiac Anesthesia, Department of Anesthesiology, Critical Care and Pain Medicine, Boston, Massachusetts, United States; c Boston Children’s Hospital, Harvard Medical School, Division of Perioperative Anesthesia, Department of Anesthesiology, Critical Care and Pain Medicine, Boston, Massachusetts, United States; d Boston Children’s Hospital, Harvard Medical School, Division of Biostatistics, Department of Anesthesiology, Critical Care and Pain Medicine, Boston, Massachusetts, United States; e Boston Children’s Hospital, Harvard Medical School, Sports Medicine Division, Department of Orthopedic Surgery, Boston, Massachusetts, United States; f Université de Montréal, Département en Neuroscience, Centre de Recherche du CHUM, Montréal, Quebec, Canada; g Massachusetts General Hospital, Harvard Medical School, Departments of Psychiatry and Radiology, Boston, Massachusetts, United States

**Keywords:** brain, cortex, nociception, neural blockade, knee, orthopedic, anterior cruciate ligament repair

## Abstract

**Significance:**

Functional near-infrared spectroscopy (fNIRS) has evaluated pain in awake and anesthetized states.

**Aim:**

We evaluated fNIRS signals under general anesthesia in patients undergoing knee surgery for anterior cruciate ligament repair.

**Approach:**

Patients were split into groups: those with regional nerve block (NB) and those without (non-NB). Continuous fNIRS measures came from three regions: the primary somatosensory cortex (S1), known to be involved in evaluation of nociception, the lateral prefrontal cortex (BA9), and the polar frontal cortex (BA10), both involved in higher cortical functions (such as cognition and emotion).

**Results:**

Our results show three significant differences in fNIRS signals to incision procedures between groups: (1) NB compared with non-NB was associated with a greater net positive hemodynamic response to pain procedures in S1; (2) dynamic correlation between the prefrontal cortex (PreFC) and S1 within 1 min of painful procedures are anticorrelated in NB while positively correlated in non-NB; and (3) hemodynamic measures of activation were similar at two separate time points during surgery (i.e., first and last incisions) in PreFC and S1 but showed significant differences in their overlap. Comparing pain levels immediately after surgery and during discharge from postoperative care revealed no significant differences in the pain levels between NB and non-NB.

**Conclusion:**

Our data suggest multiple pain events that occur during surgery using devised algorithms could potentially give a measure of “pain load.” This may allow for evaluation of central sensitization (i.e., a heightened state of the nervous system where noxious and non-noxious stimuli is perceived as painful) to postoperative pain levels and the resulting analgesic consumption. This evaluation could potentially predict postsurgical chronic neuropathic pain.

## 1 Introduction

Over 17 million surgeries are performed each year in the United States^1^ and over 300 million are performed worldwide.^2^^,^^3^ Routine surgical procedures “attack” the brain through activation of afferent nociceptive fibers following tissue damage and consequent inflammatory processes^4^ that may result in peripheral and central sensitization along with hyperalgesia/sensitization.^5^^,^^6^ The perioperative nociception and pain associated with surgery, combined with other risk factors that include premorbid psychological state,^7^ preoperative pain levels,^8^^,^^9^ and perioperative pain/nociception from the surgery itself, may lead to chronic postsurgical neuropathic pain (CPSNP; “surgical” and “operative” used interchangeably) of an inflammatory or neuropathic nature^10^ that may advance to persistent and treatment-resistant pain.^11^ Some 10% to 50% of surgeries performed in the United States annually result in CPSNP,^12^^–^^14^ with up to 5% of patients reporting severe, disabling pain 1 year following surgery.^15^ Thus, more effective intra- and postoperative analgesia and the potential to measure intraoperative pain load may contribute to the prevention of surgery-induced pain chronification.^8^^,^^16^

Neuroimaging techniques, particularly functional magnetic resonance imaging (fMRI), have illuminated our understanding of the central nervous system mechanisms associated with generation, maintenance, and chronification of pain in humans.^17^ However, the major limitation of fMRI is its inapplicability in a clinical setting, such as during surgery. Functional near-infrared spectroscopy (fNIRS), similar to fMRI, is also based on neurovascular coupling but provides long-term monitoring of hemodynamic signals while being portable, robust, and cost-effective, making it suitable for intraoperative use. It is a noninvasive neuroimaging technique that quantifies cortical concentration changes in oxygenated hemoglobin (${\mathrm{HbO}}_{2}$) and deoxygenated hemoglobin (HbR) and their total (HbT) from absorption of near-infrared light traveling through cortical tissues.^18^^,^^19^ fNIRS has been used to evaluate painful/nociceptive events,^20^^–^^30^ including patients under general anesthesia,^22^ and the effects of experimental pain following opioid analgesia.^31^^,^^32^

Based on both fNIRS and fMRI studies,^33^^–^^35^ our group has primarily focused on signals from two key brain regions during surgical procedures: (1) the primary somatosensory cortex (S1), a hallmark brain region known to be involved in central nociceptive processing and activate in response to nociception^36^^–^^38^ and (2) the prefrontal cortex (PreFC) comprising a set of functionally segregated subregions, thought to be involved in higher integration and modulation of painful/nociceptive signaling.^29^ For example, functional subregions of the PreFC, namely frontopolar PreFC /Brodmann Area 9, are found to deactivate in response to noxious electrical stimuli,^31^ while other regions such as the lateral and dorsolateral PreFC activate following evoked pain.^39^ Signals from these regions, and potentially from other cortical regions, combined with intraoperative data on the contra- and ipsilateral regions of interest (ROI) could contribute to the pursuit of outlining an objective measure of pain. One potential benefit of such a measure is the ability to appropriately time administration of medication if analgesic coverage is inadequate. Peripheral neural blockade (regional anesthesia) as part of general anesthesia is considered to limit postoperative pain^40^^,^^41^ by blocking afferent neural activity from painful surgical procedures. Poorly timed administration of analgesics or regional anesthetics and potential opioid-induced hyperalgesia^42^^,^^43^ during surgery can heighten the probability of developing an altered brain state. These events can go on to precipitate a transition from acute postoperative pain to a chronic pain state or central sensitization.^10^^,^^11^ As such, the altered brain state—a consequence of painful/nociceptive signals due to surgical trauma—may contribute to the evolution of CPSNP.^10^^,^^11^

For orthopedic surgery, such as the repair/reconstruction of anterior cruciate ligament (ACL) that is increasingly prevalent in individuals younger than 20 years of age,^44^ regional anesthesia (including femoral and/or adductor canal nerve block^45^^,^^46^) is an option for intra- and early postoperative pain control to reduce the use of opioids.^47^ However, the efficacy (i.e., percentage of successful blockade of afferent pain fibers, duration of analgesia, etc.) of a single peripheral nerve block and its effect on intraoperative response to nociception, postoperative pain, and/or analgesic consumption remains unclear.^47^^,^^48^ Hence, this study aims to expand upon our prior work using continuous-wave fNIRS to measure intraoperative pain/nociception during procedures performed under general anesthesia. We sought to derive a method for evaluating the effects of painful stimuli on cortical brain responses in PreFC and S1 for “painful” procedures during arthroscopic knee surgery. Since patients are unconscious during anesthesia, the fNIRS brain measures observed during surgical trauma (incisions, etc.) represent nociception (the detection of painful stimulus; where nociceptors are activated by a physiological process that includes transmission to neurons in the dorsal root ganglia, the spinal cord, and brain) and not pain (an unpleasant sensory and emotional experience associated with, or resembling that associated with, actual or potential tissue damage).^49^ Numerous animal^50^^–^^52^ and human^53^^–^^55^ studies support the notion that nociception can be measured in the brain under anesthesia. Yet, the complex unconscious or subconscious interactions between brain regions that occur from afferent nociceptive inputs beyond the primary somatosensory pathways (spinal cord, thalamus, primary somatosensory cortex) is currently not well understood. Furthermore, we sought to determine if fNIRS nociception/pain measures were diminished by peripheral nerve blockade with local anesthetics (i.e., regional anesthesia) in a separate subset of patients. We hypothesized that fNIRS can provide an objective measure of pain from surgical interventions (e.g., incision, cutting, etc.), while also measuring the efficacy of regional anesthesia in patients under general anesthesia.

## 2 Materials and Methods

### 2.1 Subject Eligibility, Inclusion, and Exclusion Criteria

Eligibility criteria for this study included male and female patients between 12 and 30 years underage undergoing elective arthroscopic knee surgery under general anesthesia at Boston Children’s Hospital. Exclusion criteria included inability to cooperate or understand the nature of the study, structural or genetic disorders that affect typical brain structure and function, significant medical disease, smoking history, inability to obtain reliable fNIRS measures in initial signal test, or inability to maintain head stillness for 200 continuous seconds. This protocol was approved by Boston Children’s Hospital’s institutional review board, and all participants and legal guardians assented/consented to participation in this study. This study conformed to the Declaration of Helsinki for experiments in patients related to pain.

### 2.2 Subjects

Study personnel gathered data from 24 patients (ages 12 to 25 years) who underwent arthroscopic knee surgery under general anesthesia; however, five patients were excluded from analysis; one patient had unsaved data, one patient withdrew due to medical emergency, and three patients had incomplete pre- and/or postsurgical data. Table 1 provides demographic and clinical data for the 19 patients. Eleven patients (58%) received regional anesthesia prior to surgical incision (age 17.6±3.59, six females) received regional anesthesia (nerve block; NB), while eight patients (age 18.9±2.80, five females) did not (non-NB). This decision to perform a nerve block was made by the anesthesiology team, without any predetermined factors.

**Table 1:** Demographic and clinical data. Abbreviations: M, male; F, female; R, right knee; L, left knee; Y, nerve block placed; N, nerve block not placed; ACL, anterior cruciate ligament; IT, Iliotibial band; OCD, osteochondritis dissecans.

Patient	Age (Y)	Sex	Laterality	Diagnosis	Procedure	Pain procedures (n)	Nerve block
1	17	F	R	ACL tear. Anterior horn lateral meniscus tear. Complex radial tear of the medial meniscus. Medial compartment osteoarthritis	ACL reconstruction with hamstring autograft	3	Y
				Grade II chondromalacia medial femoral condyle and medial tibial condyle			
2	19	F	L	Medial femoral condyle OCD lesion	Medial femoral condyle OCD lesion fixation and drilling	3	N
3	17	F	R	Unstable medial meniscus of the knee along with undersurface tear of the meniscus	Medial meniscus repair	5	N
4	13	F	R	ACL tear, rule out medial meniscus tear	ACL reconstruction using autologous hamstring graft. Trephination of the medial meniscus.	4	Y
5	14	M	R	ACL tear	ACL reconstruction with iliotibial band	8	Y
6	16	F	R	Knee pain s/p carticel procedure 3 years previously	Patella maltracking, scar tissue, bone spur at the notch, lateral release, chondroplasty	2	N
7	18	M	R	ACL tear, hypoplastic ACL	ACL reconstruction with hamstring autograft, notchplasty	5	Y
8	19	M	L	Complete ACL tear, lateral meniscus tear	ACL reconstruction with hamstring autograft, lateral meniscus repair	5	Y
9	22	F	R	Recurrent patellar instability	Tibial tubercle medialization osteotomy. Open medial plication	6	Y
10	14	F	R	Complete ACL tear	ACL reconstruction with hamstring autograft	5	Y
11	23	M	L	Loose body and lateral femoral condyle chondral defect	Loose body removal, lateral femoral condyle chondroplasty, partial synovectomy with plica excision, and microfracture of lateral femoral condyle	6	N
12	16	M	L	Ligament tear	ACL reconstruction under arthroscopic control	6	Y
13	25	F	L	ACL tear of the left knee	ACL reconstruction with hamstring autograft, lateral meniscus repair	6	Y
14	17	F	R	Painful plica right knee. Lateral tracking and deviation of the patella	Excision of fibrotic medial plica. Partial lateral release under arthroscopic control	2	N
15	22	M	R	Bucket-handle tear of the medial meniscus	Partial medial meniscectomy	2	N
16	17	M	L	ACL tear	ACL reconstruction, femur fixation, screw in tibia	11	Y
17	19	F	L	ACL tear	ACL reconstruction with bone-patellar-bone autograft using a 4×12 mm continuous loop endobutton suspensory fixation on the femur and a 9×23 mm BioComposite screw in the tibia	9	Y
18	21	F	R	Anterior knee pain, proximal tibiofibular joint instability	Plica excision, proximal tibiofibular joint reconstruction using semitendinosus allograft	12	N
19	16	M	R	ACL tear	ACL reconstruction with hamstring autograft	10	N

### 2.3 Anesthetic Technique

All patients underwent general anesthesia according to routine practice. Anesthetic data for each patient are shown in Table S1 in the Supplementary Material. Drug dosages are presented here as median [range]. Briefly, all patients were premedicated with 2-mg midazolam IV and propofol (median 200 mg [150 to 300 mg]) used for induction of anesthesia in 18 patients (95%). In one patient (#13), the induction dose of propofol was not recorded. Fentanyl (median 100 g [100 to 250 mg]) was administered as part of the induction in all but one patient who was given sufentanil (#2). Anesthesia was maintained with an inhalational agent, predominantly sevoflurane, and supplemented with a propofol infusion in 12 patients (47%). The airway was managed with a laryngeal mask airway and spontaneous ventilation; neuromuscular blockade was not employed. For intraoperative analgesia, hydromorphone (median 0.6 mg [0.3 to 1.6 mg]) was the opioid of choice and acetaminophen (650 mg IV) was administered intraoperatively to all but one patient. Ketorolac (median 30 mg [18 to 30 mg]) and diazepam (median 2.5 mg [2.5 to 7.5 mg]) were each administered to approximately half the patients. The acetaminophen, ketorolac, and diazepam were generally given toward the end of the surgery. Antiemetic prophylaxis included ondansetron (4 mg) in all but one patient (#17), dexamethasone (median 4 [4 to 8] mg) in all but three patients (#s 1, 3, 16), and a scopolamine patch (1.5 mg) was applied in two patients (#s 6, 13).

An adductor canal peripheral nerve block with ropivacaine (0.2% or 0.35%) was performed under ultrasound guidance on the ipsilateral limb in 11 (58%) patients. Nerve block was administered based on the clinical team’s recommendation following standard clinical evaluation and the patient’s consent. Details for the regional anesthesia are presented in Table S1 in the Supplementary Material. A catheter for infusion of local anesthetic was placed in one patient (#17) but the infusion was only started 6 h postoperatively. In one patient, a lateral femoral cutaneous nerve block was added to the adductor canal block. Dexmedetomidine and clonidine were added to the ropivacaine in one (#7) and two (#s 5, 9) patients, respectively.

Local anesthetic (0.25% bupivacaine with 1:200,000 epinephrine) was injected by the surgeon in 16 patients (84%), either immediately prior to incision (n=9) or at the end of surgery (n=7). Of the 11 patients that received a peripheral nerve block, local anesthetic was injected by the surgeon in 10 cases either with incision (n=8) or at the end of surgery (n=2). For patients that did not receive a nerve block (n=8), local anesthetic was injected by the surgeon at the end of the procedure in six patients.

### 2.4 Surgical Procedure

Surgical procedures were performed according to standard practice and are shown in Table 1.^56^^,^^57^ Each subject laid flat in the supine position for the entire duration of the study. Surgical technique involved skin incision and dissection of cutaneous tissue to create portals for insertion of arthroscopes and related instruments, shaving of torn ligamentous tissue, cutting of hamstring, iliotibial band, and/or patellar bone for autografts, drilling, and suturing of surgical incisions. We chose to define “skin incisions” as the painful events, i.e., P1 and P2. As with any surgical procedure (injections, scraping, nerve block applications), other processes may be painful. Notwithstanding this decision, we believe that fNIRS will be able to detect any painful procedure. A verbal rating scale (VRS) was used to evaluate pain intensity (0 being no pain; 10 being the worst pain imaginable) in each patient just prior to surgery, shortly after arrival in the post-anesthesia care unit (PACU) and just prior to discharge from the PACU.

### 2.5 Data (fNIRS) Acquisition

**Fig. 1:**
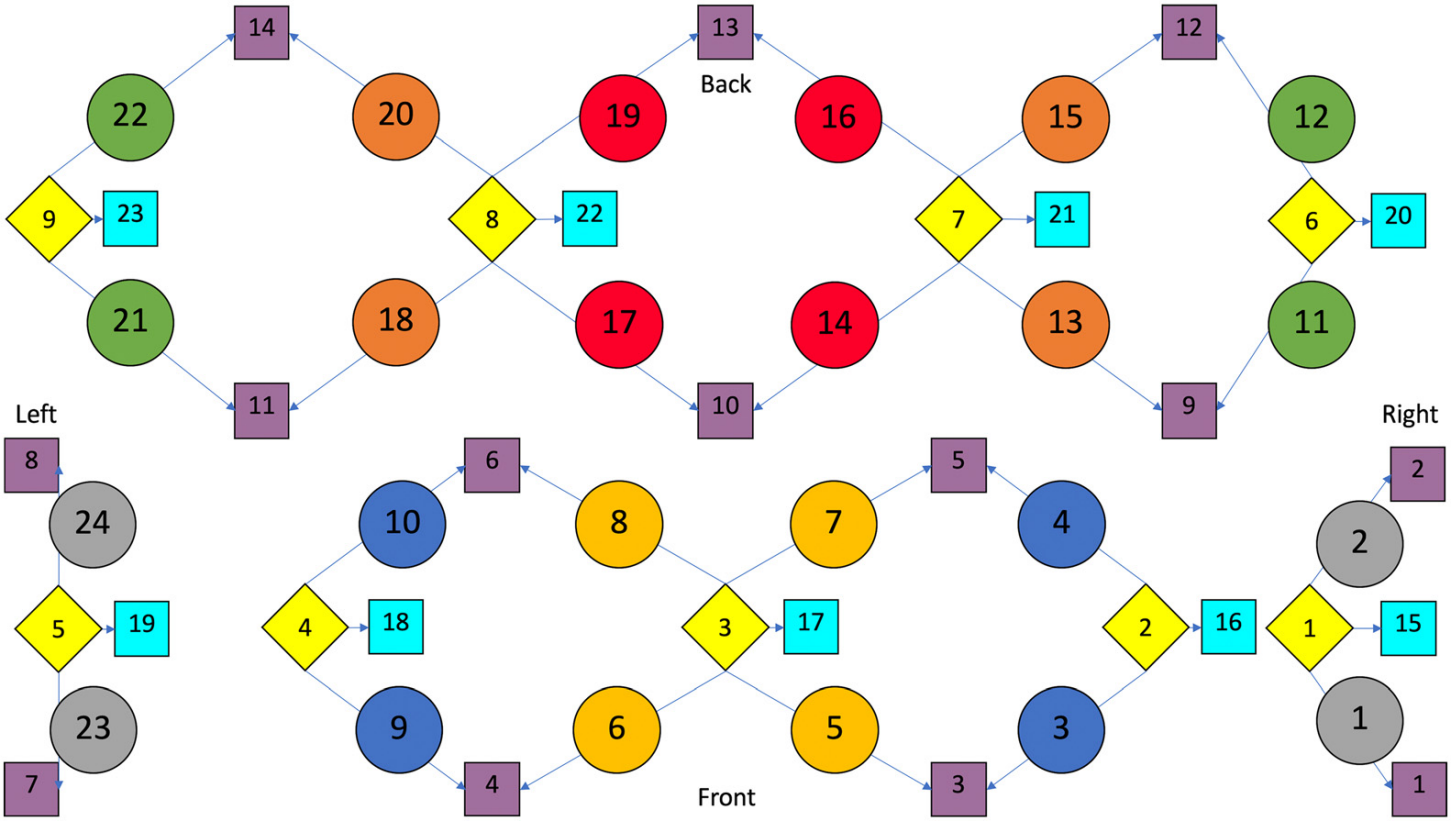
fNIRS node map. The nine sources marked in yellow are detected by the 14 regular detectors in purple and the nine short-source detectors in blue. Black arrows represent the 3 cm between the sources and the long-separation detectors while the red arrows show the 0.8 cm between the sources and the short-separation detectors. The 24 connections between the sources and the detectors are the channels that feedback the concentrations of oxygenated (${\mathrm{HbO}}_{2}$), deoxygenated (HbR), and total (HbT) hemoglobin to the end user. The front 12 PreFC channels are separated into the lateral (silver), pre-lateral (blue), and medial (yellow) groups while the back 12 somatosensory cortex (S1) is split into the superior (red), central (orange), and inferior (green) S1 groups.

**Fig. 2:**
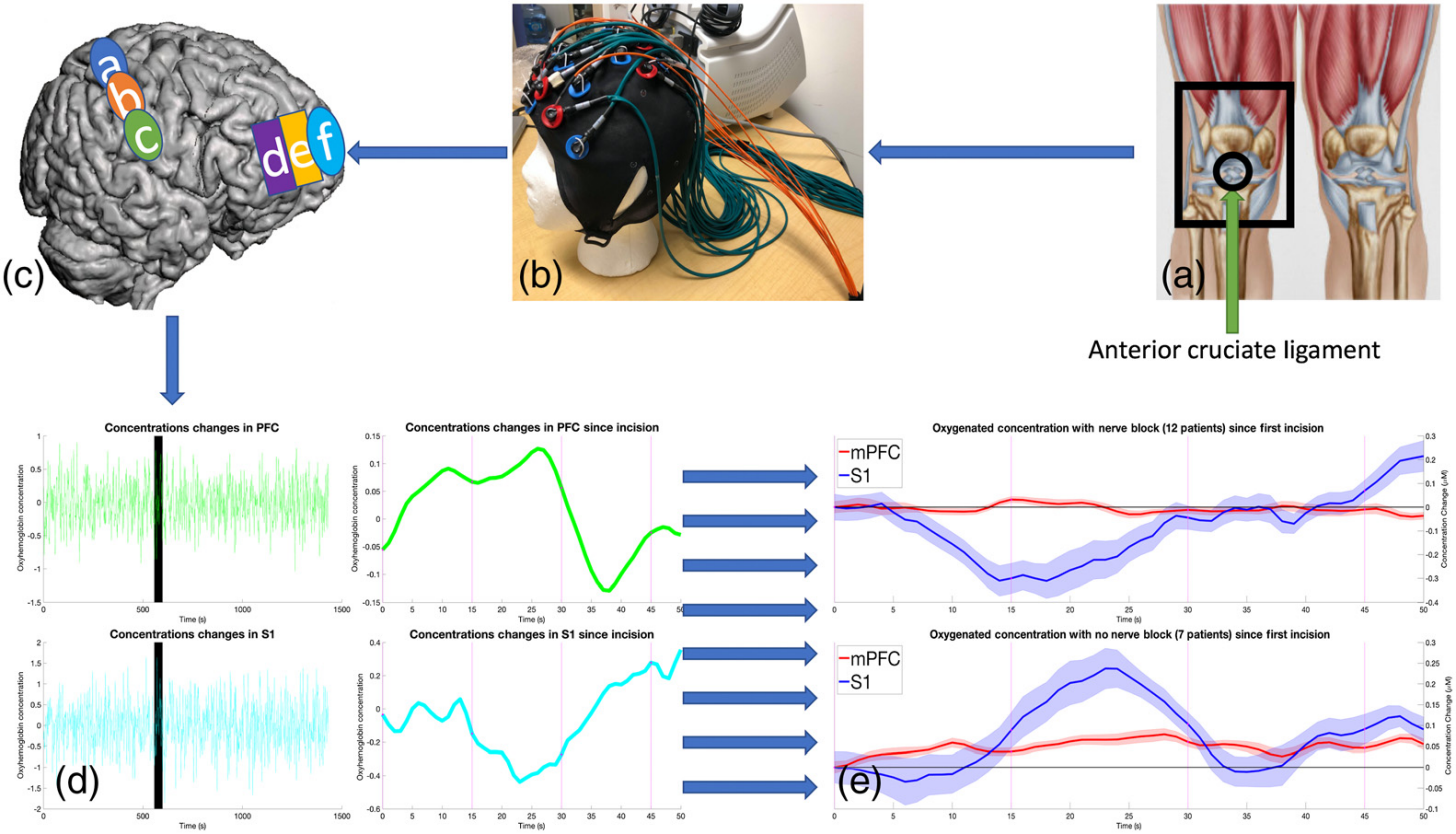
fNIRS timeline. Each patient has one of their knees operated on [as shown in (a)^59^]. Using the fNIRS cap in (b), concentration readings are obtained from the 24 nodes attached to the head. These nodes observe four concentrations in the six ROIs shown in (c) (a, superior S1; b, central S1; c, inferior S1; d, lateral frontal cortex; e, lateral polar frontal cortex; f, mPFC). As the opposite cortex is observed and since the right knee is operated on, we look at the 12 channels in the left cortex (7 to 12 in the frontal PreFC and 19 to 24 in the rear in Fig. 1). ${\mathrm{HbO}}_{2}$ and HbR are recorded for 25-min after surgery starts [see (d) for the average oxyhemoglobin of the six relevant filtered channels for PreFC and S1, with the standard error represented each side of the error bars]. An incision was performed at the start of the section of time marked in black and continued through the black bar’s duration. A sliding window of 10 s in 1 s increments was then calculated for the minute after the incision. This period of time is then examined for trends in the patient (no nerve block was administered to this patient). When all the patient data have been collected, the average oxygenated hemoglobin of the patients who did receive nerve block and those who did not were calculated and plotted along with a running error bar over time as shown in (e).

Data were collected using a multichannel fNIRS system at 690 and 830 nm wavelengths and 25 Hz sampling frequency (TechEn Inc., Milford, Massachusetts, CW7 System). The changes in hemoglobin concentration of oxygenated (${\mathrm{HbO}}_{2}$), deoxygenated (HbR), and total hemoglobin (HbT) within the cortical areas were measured based on the modified Beer–Lambert law. We have previously described techniques of using fNIRS for measuring of pain/nociception under anesthesia.^22^ A similar approach was taken here. A custom probe with nine sources and 14 long-separation detectors (placed ∼3  cm from the source) and nine short-separation detectors (placed 0.8 cm from the source)^58^ were used (see Fig. 1). This probe was designed using an EEG cap with a 10–20 layout to include 24 channels covering the bilateral PreFC and the bilateral somatosensory cortices [see Fig. 2(b)]. We specifically evaluated six brain ROIs: the PreFC consisting of three ROIs: (1) the medial polar frontal cortex (mPFC), (2) lateral polar-frontal cortex (lPFC), (3) lateral pre-frontal cortex (lateral Pre-FC) and the bilateral somatosensory cortex (S1) also made up of three ROIs: (4) superior S1, (5) medial S1, and (6) inferior S1.

Prior to entering the operating room, the probe-containing cap was securely placed on patients’ heads. The optodes were secured using custom designed washers to maintain contact (see Fig. S1 in the Supplementary Material). A smaller EEG cap with the same probe layout was used for pediatric patients as needed. Data quality and optode positions were confirmed with a 10-min baseline scan in the preoperative setting. There were two steps of quality control, one during data acquisition and one during postprocessing. After setup and before the beginning of the scan, the channels were visually inspected. Good contact between the scalp and the optode was confirmed by (1) acceptable power intensity (as indicated by CW7 software) and (2) a visible heart rate in all the channels, including short-separation channels. After which, the fNIRS data were continuously collected, beginning at anesthetic induction, and concluding following final suturing at the end of surgery. During this period, one research personnel was responsible for monitoring fNIRS data acquisition quality, while another was responsible for noting the time and type of surgical procedures; for instance, the initial incision was noted as “incision,” arthroscopic scraping was noted as “scraping,”and so on. A summary of fNIRS signal acquisition and data analysis for a single patient is shown in Fig. 2.

### 2.6 Preprocessing

Raw signals and their power spectrum were visually inspected using Homer2 toolbox. If at least two out of the four channels within a cortical region (lateral PreFC, medial PreFC, and somatosensory cortex) exhibited a visible heart rate component in the raw time series and a discernible peak at the cardiac frequency range of the power spectrum, that region qualified as “good” data. If the raw time series exhibited a heart rate component, but the data were noisy, it was classified as “average” data. If the raw time series did not show a visible heart rate, the data were too noisy, and the power spectrum did not show a discernable heart rate component, then that data were considered “poor” data. This classification was performed for every subject. Subjects with at least two cortical regions with “average” or more data were included in the next steps of the analysis. Raw fNIRS time series data were then preprocessed using scripts written by our group in the MATLAB R2018b platform. The raw fNIRS signal was converted to optical density and corrected for head-motion using a wavelet-based algorithm.^60^ Motion-corrected fNIRS data were then bandpass filtered at 0.01 to 0.3 Hz and converted to concentration data using the *hmrOD2conc* function in the Homer2 toolbox.^61^^–^^63^ A linear temporal regression of the hemoglobin (${\mathrm{HbO}}_{2}$, HbR, and HbT) time series of each cortical channel was performed using the nearest short-separation (physiological) channel recording as a nuisance regressor.^23^ The time series of long-separation channels was the dependent variable, and the time series of the nearest short-separation channels was the independent variable/regressor, as shown in Eq. (1):

$$Y_{\text{long-separation}}=b_{0}+b_{1}X_{\text{nearest short-separation}}.$$ (1)

This was performed for every long-separation channel (n=24). The residual hemoglobin time series from temporal regression was then fitted using a third-order polynomial to remove nonlinear drifts. Lastly, the data were corrected for linear drifts and converted to microMolar (μM) units.

### 2.7 Data Analysis

After preprocessing, the time series for ${\mathrm{HbO}}_{2}$, HbR, and HbT of the 12 channels on the hemisphere contralateral and the 12 channels on the ipsilateral hemisphere to the operated-on knee were retrieved (see Fig. 1 for diagram of channels). The ${\mathrm{HbO}}_{2}$ time series of these adjacent channels in each cortex were averaged within the contralateral and ipsilateral ROIs to form a single time series for the six different ROIs in every patient (see Fig. 2 for an illustration of this process for a single patient).

### 2.8 Rolling Average Concentration Change

**Fig. 3:**
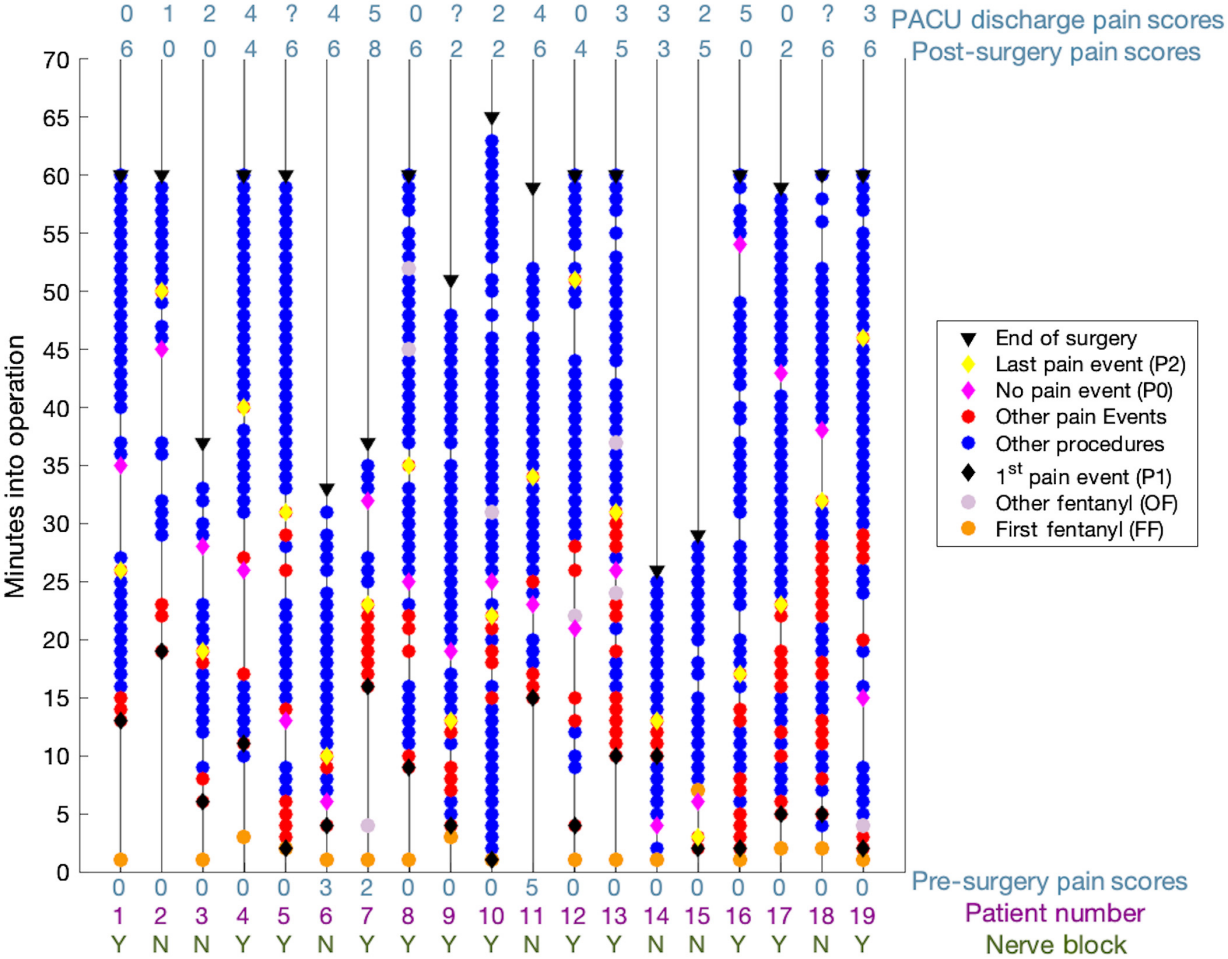
Timeline for surgical events during anesthesia. Presurgical pain scores for each patient were ranked on a 0 to 10 VRS scale (0 being no pain; 10 being worst pain imaginable). After presurgery, NB patients are marked with a Y and non-NB patients are marked with an N. The first incision, representing the first instance of pain in the patient (P1), occurs in the minute marked with the black diamond. The 60 s that are furthest away from an event (painful or nonpainful) are marked with a magenta diamond (P0) and the last incision is marked with a green diamond (P2). Other procedures which are painful in nature happen within the minute increments marked with red circles, while nonpainful procedures are marked with blue circles. Surgery lasted between 25 and 65 min for each patient. Another VRS pain score was taken in the recovery room postsurgery, and a final score after being discharged from the PACU.

Concentration changes in these six ROIs were investigated at three different time points: no procedure (P0; i.e., no surgival interventions during this time), first incision (P1), and last incision (P2). P0 was defined as the 60-s intraoperative epoch in which patients did not receive any procedure. This metric was individually determined for each patient by selecting the longest possible lapse of time between any procedure (minimim 2 min; maximum 9 min; average 4.5 min). This was because there was not enough duration before any kind of surgical intervention was made. P1 was defined as the 60-s epoch following the first incision, and P2 was defined as the 60-s epoch following the last incision. All epochs were normalized to the mean at the time of observation. A timeline of the surgical procedures including the P0, P1, and P2 events in each patient is shown in Fig. 3.

Next, a standardized 10-s sliding window with 1-s increments was calculated for the 60 s during P0, P1, and P2. The area for the absolute standardized rolling average curves was computed for the six ROIs in each patient using the *trapz* function in MATLAB. A mixed ANOVA approach was utilized to protect against type I errors due to multiple comparisons. This helps to identify significant differences in the area under the curve measure of P0, P1, and P2 procedures in NB versus non-NB groups. Statistically significant effects were obtained at the significance level of p<0.05. A Benjamini–Hochberg false-discovery rate (FDR) correction for multiple comparison problems (at α=0.05) was also utilized (PMID 28740688).

### 2.9 Functional Correlation

Between the 15 unique ROI–ROI connections for a given epoch, we calculated Pearson’s r correlation coefficient for P0, P1, and P2 conditions. These average changes in correlation over time were grouped into the PreFC, S1, and PreFC/S1 regions (by averaging all channels within the contralateral hemisphere) for NB and non-NB patients. This was computed using a sliding window correlation approach (see Fig. 7 for an example of this). A mixed ANOVA approach was used to identify significant differences in the functional correlation measures and dynamic functional correlation measures of P0, P1, and P2 procedures in NB versus non-NB groups and over time. Any statistically significant effects were recorded at significance level of p<0.05 with a Benjamini–Hocberg FDR correction for multiple comparison problem (at α=0.05).

## 3 Results

### 3.1 Pain Events With and Without Nerve Blockade: Dynamic Hemoglobin Concentration and Correlation Values

The values of $\Delta [{\mathrm{HbO}}_{2}]$, Δ[HbR], and Δ[HbT] on the contralateral and ipsilateral sides of the brain corresponding with affected knee showed significant relations between NB and non-NB groups, and between the pain events P1 and P2, and the control period P0. Between the 18 combinations of hemoglobin concentration, observed periods of time (P0, P1 or P2), proximity to the knee and the six ROIs, we observed the following traits indicative of the presence of nerve block and the presence of pain. Below we note the three major findings.

#### 3.1.1 Large contralateral $\Delta [{\mathrm{HbO}}_{2}]$ increases following a painful event in non-NB patient group

**Fig. 4:**
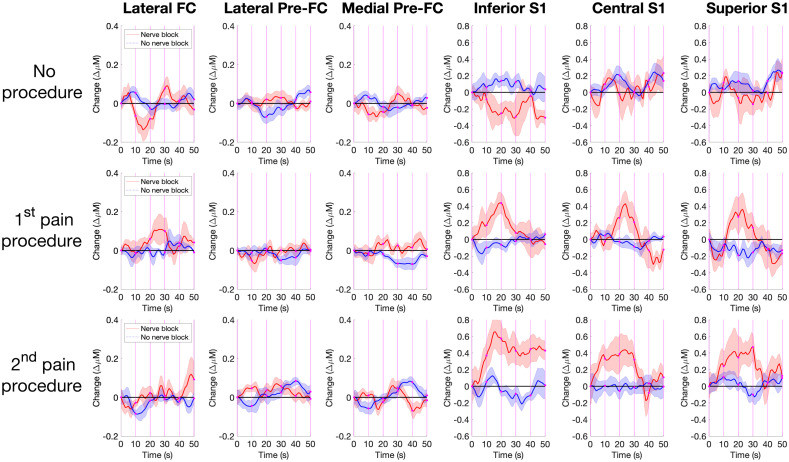
Mean concentration over time. Rolling averages and standard errors for the standardized contralateral oxygenated hemoglobin concentration ($\Delta [{\mathrm{HbO}}_{2}]$) are observed for six relevant ROIs; lateral polar frontal cortex (lateral FC), lateral Pre-FC, medial Pre-FC, inferior S1, medial S1, and polar S1. These are recorded for the minute where no procedure occurs (P0), the minute after the first recorded incision takes place (P1), and the minute after the last recorded incision takes place (P2). Average concentrations of patients who were given nerve block (NB) are in red and patients not given nerve block (non-NB) are in blue. Correlations are taken every 10 s at the points marked in magenta and are shown in Fig. 7.

The time series graph in Fig. 4 shows the average contralateral $\Delta [{\mathrm{HbO}}_{2}]$ and its standard error over the six ROIs. (The median figure is shown in Fig. S2 in the Supplementary Material.) No significant relationships are observed in the PreFC ROIs during any event, with all $\Delta [{\mathrm{HbO}}_{2}]$ concentration levels staying within ±Δ0.2  mM of the baseline. The most notable finding in concentration difference is the poststimulus increase in non-NB patients. This reaches an average amplitude peak of +Δ0.41 mM after around 20 s in P1 and a ≥30  s epoch after P2 (this peak becomes the new baseline in InfS1 while this signal declines to the baseline in CentS1 and SupS1). Conversely, for patients without a nerve block, an overall decrease in $\left[{\mathrm{HbO}}_{2}\right]$ was observed within the first 5 s in inferior S1. The changes in $\left[{\mathrm{HbO}}_{2}\right]$ observed in S1 were not seen in data obtained in the absence of painful procedures. This shows a repeatable measure to detect the presence of a pain event for patients without a nerve block (and potentially with one).

#### 3.1.2 Areas between $\Delta [{\mathrm{HbO}}_{2}]$, Δ[HbR], and Δ[HbT] and the x axis in some contralateral S1 ROIs are statistically significant between NB and non-NB groups

**Fig. 5:**
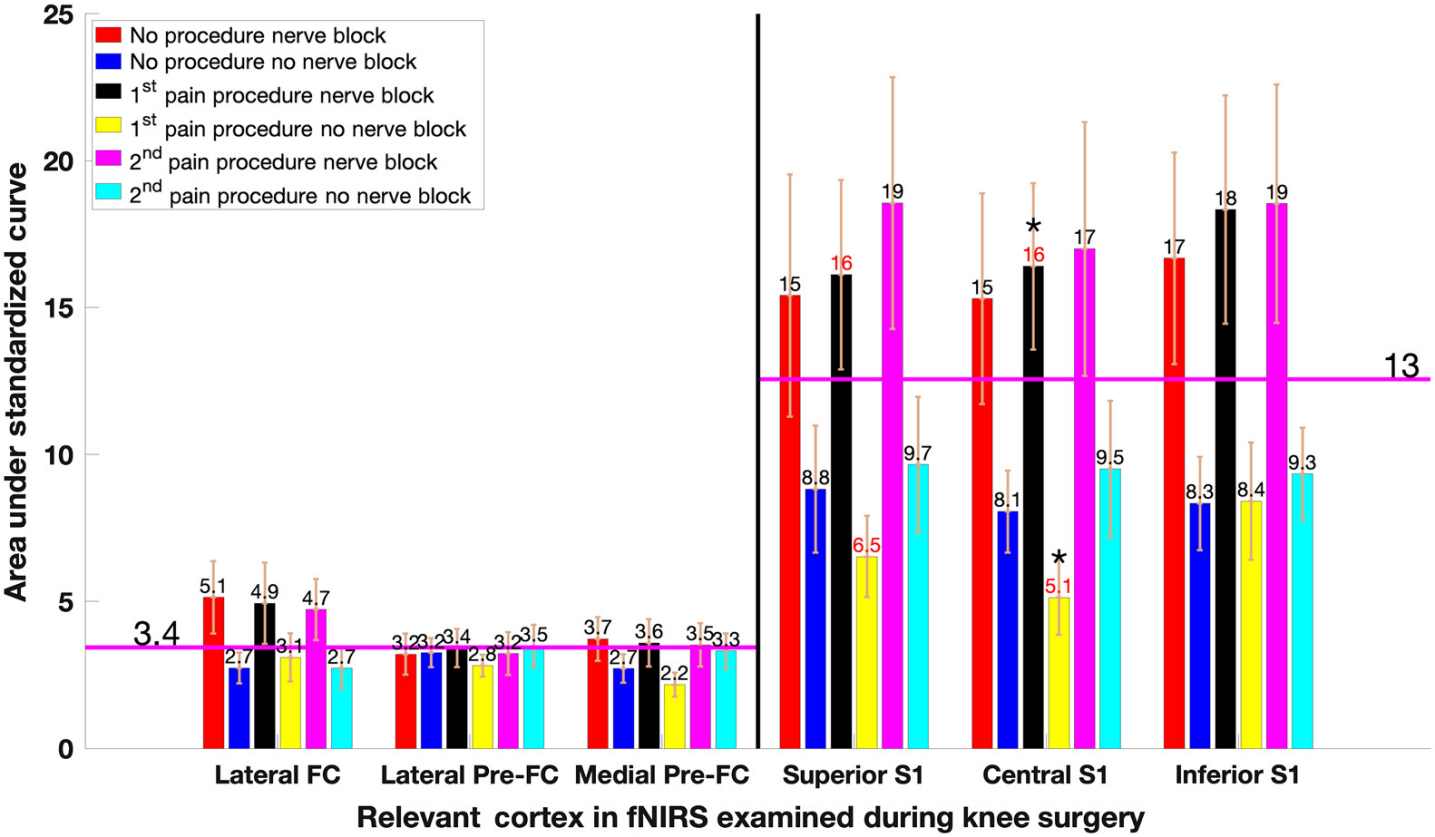
Bar chart of area under standardized curves for 12 channels. The average area captured between the X axis baseline and the NB and non-NB curves (see Fig. 4) for each of the six ROIs is shown for P0, P1, and P2. The labels on each bar in magenta show the total area captured with the error bar representing the standard error of the group. The two magenta lines show the total mean for the PreFC and S1, respectively, for their six areas captured by the curve. A two sampled t-test was conducted between the nerve and non-nerve blocks for $\Delta [{\mathrm{HbO}}_{2}]$ (see Table S3 in the Supplementary Material). Numbers in black show that the null hypothesis has been accepted such that both vectors come from normal distributions with equals means while numbers in red show the nerve block and non-nerve block vectors reject the null hypothesis. Bars marked with a (*) are statistically significant after FDR correction at α=0.05.

Absolute areas between contralateral and ipsilateral $\Delta [{\mathrm{HbO}}_{2}]$, Δ[HbR], and Δ[HbT] and the x axis are on average four times larger for S1 ROIs than PreFC ROIs and twice as large for NB groups than non-NB groups (see Fig. 5 for a visual representation of contralateral $\Delta [{\mathrm{HbO}}_{2}]$). Across the 18 observed combinations in Table S2 in the Supplementary Material, there are 10 relations between the NB and non-NB groups that are statistically significant under a mixed model ANOVA approach. Of these, eight of these are in the contralateral S1 region. Notably, all three observed time periods (P0, P1, and P2) are significant for contralateral Δ[HbR] in central S1. The result for contralateral P1 $\Delta [{\mathrm{HbO}}_{2}]$ and Δ[HbR] is also shown to be statistically significant after FDR at α=0.05.

**Fig. 6:**
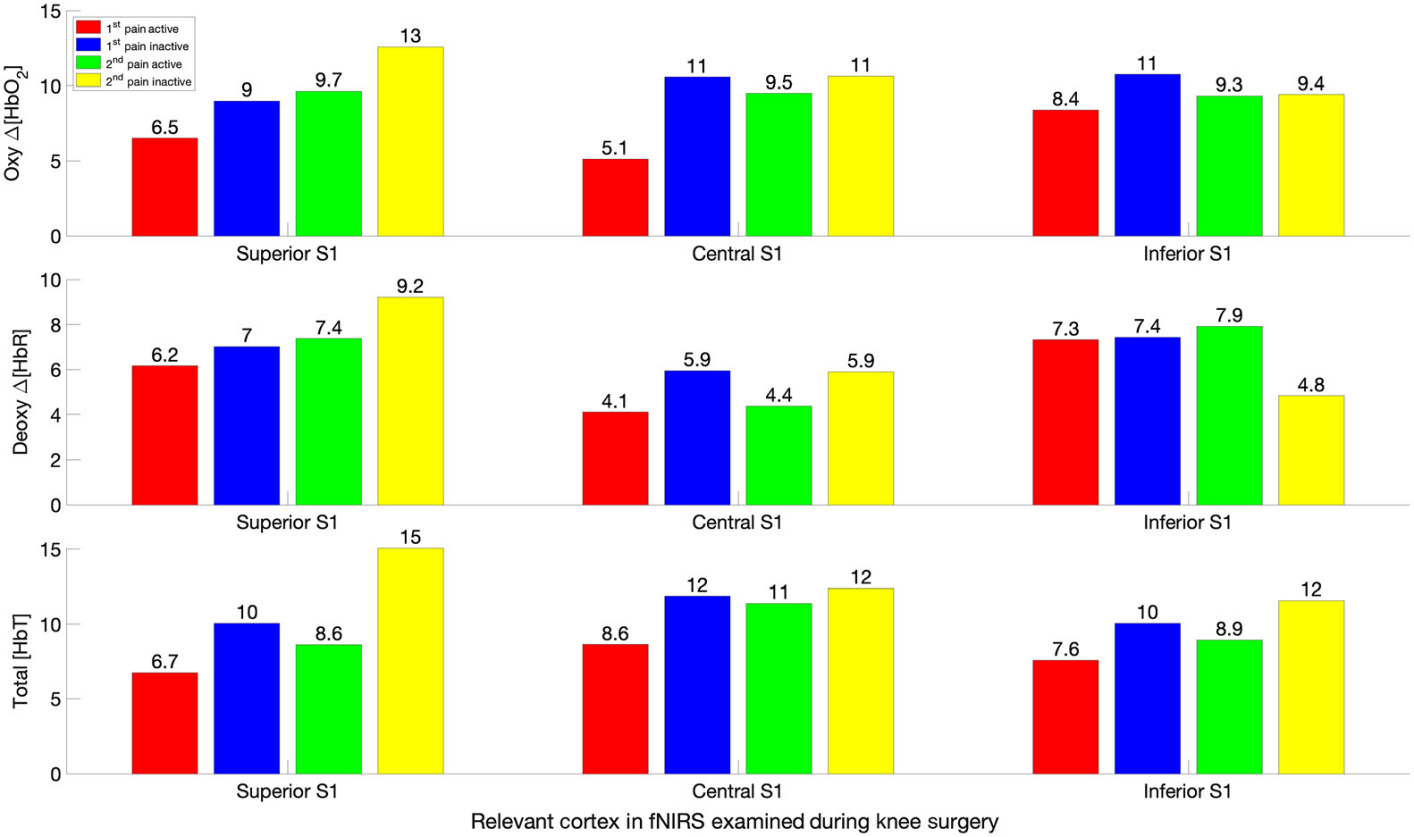
Activated versus inactivated areas under the curve for P1 and P2. Activation on the contralateral slide is compared against the inactivation on the ipsilateral side. These area between the curve and the x axis for non-NB patients are compared between the P1 and P2 periods. The area for the activated side was smaller than the inactivated side except for changes in deoxyhemoglobin in inferior S1 during P2.

These significances provide crucial information for showing the presence of a nerve block within the patient based on the findings thus far, especially early on into surgery observing contralateral P1 CL $\Delta [{\mathrm{HbO}}_{2}]$ and Δ[HbR]. Finally, a strong repeatability measure is observed in the non-NB groups for pain procedures in ipsilateral Δ[HbR] for the superior S1 ROI (underlined in Table S2 in the Supplementary Material).^64^^–^^66^ These concentrations are shown to be consistent across patients during pain events, and a moderate resemblance during the recorded nonpain event P0. Differences between absolute areas under the curve for contra- and ipsilateral ROIs for P1 and P2 in non-NB are shown in Fig. 6.

#### 3.1.3 There are three significant differences between NB and non-NB groups and one significant difference over time that are repeated over contralateral and ipsilateral $\Delta [{\mathrm{HbO}}_{2}]$

**Fig. 7:**
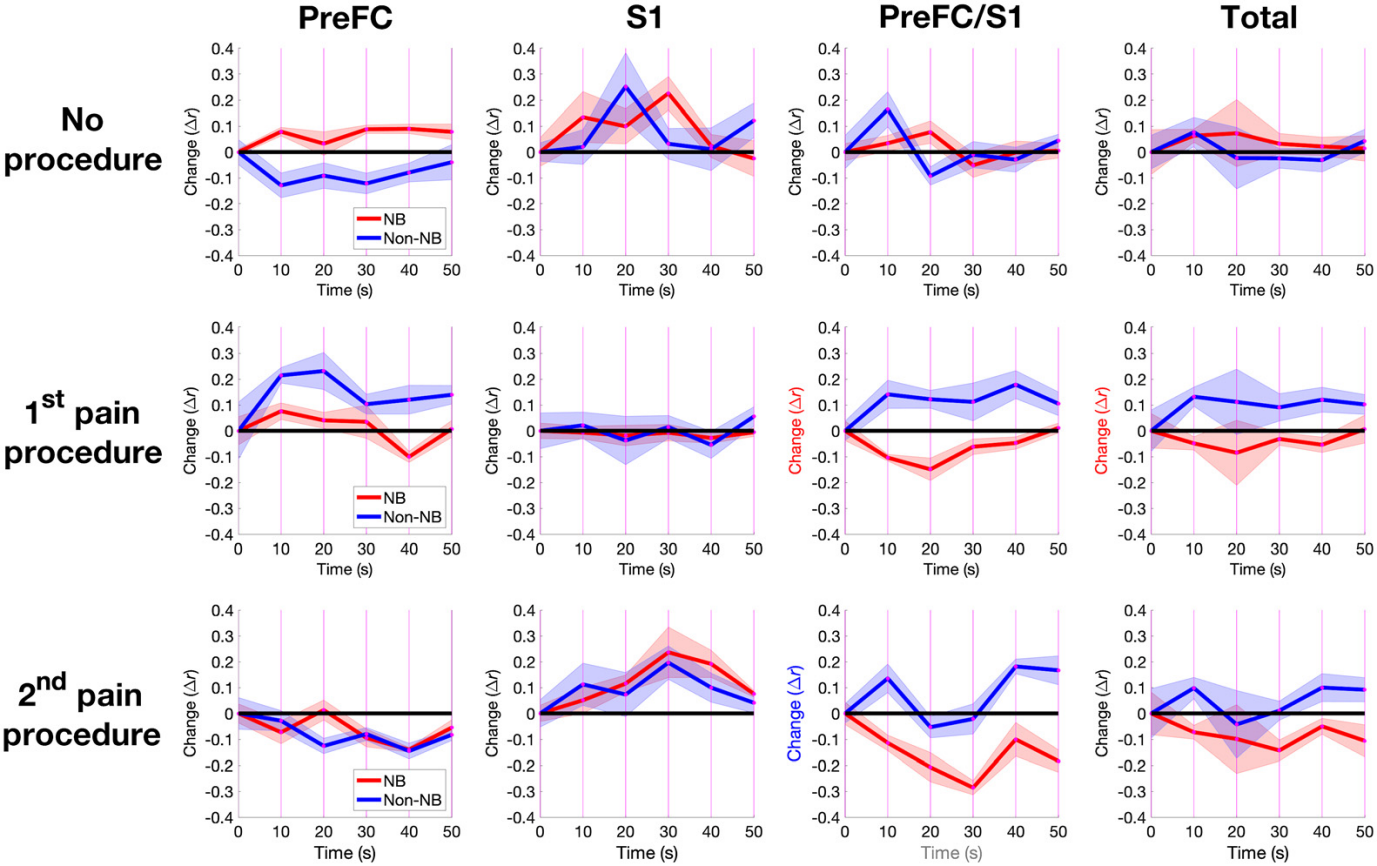
Average correlation over time. The average standardized correlations of $\Delta [{\mathrm{HbO}}_{2}]$ for all channels within PreFC, S1, between PreFC/S1 and their totals were calculated using a 10-s window incremented by 1 s. Only the correlation measures at 10-s windows (0,10,20,30,40,50) are shown by the magenta lines in Fig. 4 along with standard error of mean. Regions with significant effects of time on $\Delta [{\mathrm{HbO}}_{2}]$ are indicated the x axis label “time (s)” is highlighted in red and in blue if it remains significant after FDR, with significant effects of NB-time interaction indicated using the y axis label “change (r).”

Correlation analyses were performed to determine if interactions between ROIs could provide further insights to the cortical hemodynamic response to pain procedures. Correlations between ROIs were shown to be robustly positive in PreFC regions, weakly positive in S1 and very weakly negative in the PreFC/S1 overlap. Figure 7 shows the average changes in correlation over 10-s intervals for the PreFC and S1 regions, their overlap PreFC/S1, and across all correlations for $\Delta [{\mathrm{HbO}}_{2}]$. This graph shows four relations that are statistically significant (p<0.05), with (a) and (b) remaining statistically significant after FDR. (a) The effects of time in the PreFC/S1 overlap during P2. (b) The correlations between NB and non-NB groups in the PreFC/S1 overlap during P2. (c) The correlations between NB and non-NB groups in the PreFC/S1 overlap during P1. (d) The correlations between NB and non-NB groups in the total of all correlations during P1.

Table S3 in the Supplementary Material shows that observations (a) to (d) is repeated across the ipsilateral side for $\Delta [{\mathrm{HbO}}_{2}]$; however, while only (b) is significant after FDR (α=0.05) at on the contralateral side, (b) to (d) are also significant on the ipsilateral side in this way. (b) and (c) show a repeated measure between NB and non-NB groups that is not present during the control period P0 that holds for all pain events to the patients.

## 4 Discussion

Our results in Sec. 3 demonstrate the potential utility of fNIRS measures of evoked pain/nociception during ongoing surgical anesthesia. These results build on prior work evaluating the acute response to a painful procedure under surgery.^22^ Here, we further evaluate the utility of fNIRS in patients undergoing ACL repair with and without neural blockade. Our principle findings include: (1) significant changes in fNIRS signal during incisional procedures; (2) significant differences in fNIRS signals in patients under general inhalational anesthesia with and without neural blockade; and (3) reproducibility of the findings at different times of surgery following a traumatic surgical event (i.e., incision/cutting). While nociceptive activation of the central nervous system occurs with deep general anesthesia, clinical responses to pain may not be detected, as determined by functional imaging and neurophysical monitoring.^67^^,^^68^ Thus, there is a need to provide an ongoing objective measure of nociception during surgery.

### 4.1 fNIRS Signal Changes to an Incisional Procedure

We obtained continuous bilateral recordings from three brain regions in patients under general anesthesia: S1, mPFC and LPFC as shown in Sec. 3. We interpret the results from these recordings as: (1) fNIRS can measure a surgical incision event—the cortical response occurs within 5 s of the incision, clearly within the time-domain of fNIRS response;^69^ (2) even with a regional nerve block (although not evaluated for efficacy due to administration after patients were unconscious), fNIRS measurements of changes in $\left[{\mathrm{HbO}}_{2}\right]$ are still observed, albeit opposite to those observed without a nerve block (discussed below), suggesting that a painful event can be temporally evaluated; and (3) the measure is repeatable even in the context of apparent differences in assumed fentanyl plasma concentrations.

Taken together, we have deduced a number of indicators (fNIRS response time) locked with incision presumed to activate nociceptor fibers with an incision. This is dependent on measures of $\left[{\mathrm{HbO}}_{2}\right]$ determined during the surgical procedure. As such, using fNIRS, it may be possible to provide a signal for pain/nociception over the course of the surgical procedure.

### 4.2 fNIRS Measures of the Response to Surgical Trauma: Is this Nociception/Pain?

Here, we discuss three aspects of why we consider our fNIRS measures to likely be measures of pain/nociception. We are careful to use the term “pain” here, since it normally refers to conscious perception (see definition by the International Association for the Study of Pain).^70^ We are confident that we are measuring nociception, since (1) the fNIRS signals are observed and temporally associated with the incision; (2) the brain regions are associated directly and indirectly, including higher order, with the processing of nociception; (3) the use of opioid analgesics (i.e., fentanyl) diminishes the fNIRS signal, which is presumably a result of activation of the nociceptive pathways. The nerve blocks may also contribute to this understanding.

Tissue damage activates nociceptors via direct damage to pain fibers.^71^ Once initiated, inflammatory processes promote ongoing pain via activation of primary afferent neurons and may produce peripheral sensitization (enhancement of signal transmission to painful or nonpainful stimuli).^72^ Such changes may induce central sensitization because of the ongoing afferent barrage from the initial damage or subsequent tissue damage.^73^ To date, few studies have shown that attempts at complete neural blockade of afferent pathways can be accomplished to prevent central sensitization. This is relevant for a number of reasons, including the ideas that central sensitization may lead to increased postoperative pain,^74^ which may potentially be the initial harbinger of pain chronification.^75^

As noted above, nociceptive activation of the central nervous system may occur with deep general anesthesia—although clinical responses may not be detected —and nociceptive activation persists under general anesthesia in healthy volunteers in the brain^67^ and spinal cord.^66^ Unless there is a complete, successful nerve block, nociceptive afferent information will reach the cortical regions of the brain^76^—as is evidenced from our group’s findings, using fNIRS, that cortical activity is present under general anesthesia in patients undergoing cardiac ablation.^22^ Such data are corroborated by findings in anesthetized rats, using anesthetic agents, such as propofol,^77^ and in patients under propofol sedation for colonoscopy.^24^

### 4.3 Are the Areas (S1, mPFC, lPFC, and Lateral Pre-FC) Used in our fNIRS Signal Measures Involved in Pain/Nociceptive Processing?

The primary somatosensory cortex is well defined to be involved following primary nociceptive activation in animal and human studies.^78^^,^^79^ Usually, the activation is somatotopically organized based on the human homunculus^80^^,^^81^ but may be altered by nerve blockade^82^^,^^83^ or ischemic anesthesia.^84^ The PreFC has a major role in pain processing,^85^ and we have previously summarized the role of the polar frontal cortex in pain processing.^29^ The lateral frontal cortex included the ventral- and dorsolateral prefrontal cortex has also been documented to be activated following painful stimuli.^86^ Taken together, while some of these regions may have more direct involvement in nociceptive pathways, they are nonetheless all involved through functional connectivity as part of the pain connectome.^87^

Opioids are potent analgesics for acute pain^88^ and are frequently administered as part of the anesthesia protocol because inhalational anesthetics are not potent analgesics.^89^^,^^90^ In our patient group, the opioid fentanyl (50, 100, or 150  μg), which acts on μ-opioid receptors, was administered intravenously at the beginning of the anesthetic procedure (n=17), and in some cases (n=6), repeated. Using the intravenous route, the time to maximum concentration is around 5 min, with the analgesic effect being delayed by around 2.5 min and lasting, on average, over 2 h.^87^^,^^91^ One dose of intravenous 100  μg fentanyl has an equivalent analgesic effect of 10 mg of intravenous morphine. Our prior work has indicated a dose-dependent effect of oral morphine (10 mg) to a painful stimulus.^31^ While opioids may produce their analgesic effects by peripheral and central mechanisms, complete blockade at clinical doses used in the anesthetic regimen may not be possible, although fentanyl^73^ and remifentanil,^92^ for example, suppress brain activation as measured by positron emission tomography with 1.5  μg/kg in healthy volunteers and EEG measures in rats.^93^

**Fig. 8:**
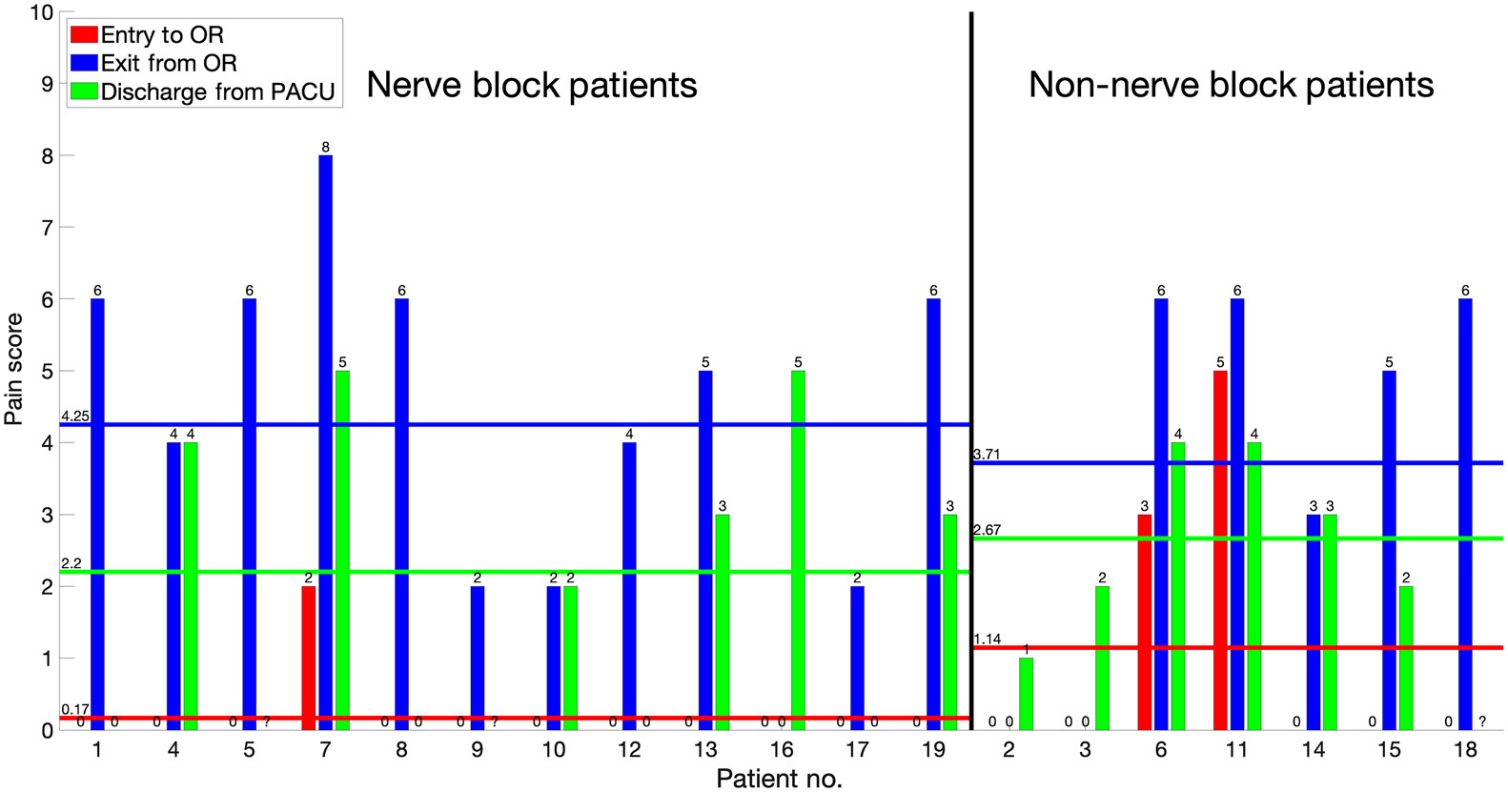
Pain scores. Patients are asked to self-report pain levels on a VRS scale of 0 to 10 (0 being no pain felt; 10 being worst pain imaginable) at three times during their visit: presurgery, postsurgery, and after discharge from the PACU. Patients are separated into NB and non-NB groups, and the average of each group is shown on top of each color-coded line on the left-hand side. Differences in scores pre- to post-surgery ranged from no change noticed in patients 2, 3, and 16, to a pain score increase of 6 observed in patients 1, 5, 7, 8, 18, and 19. The mean of the change in pain scores pre- to postsurgery was 3.53 with a standard error of 2.7 showing that in the average surgical window of 51 min, a large increase in pain is felt, increasing cases of “no pain” (VRS 0) to “mild pain” (VRS 1-3), and “mild pain” to “moderate pain” (VRS 4-6).^95^

We observed an increase in the fNIRS response at the last incision. We suggest that there are two mechanisms that may be at play: (1) hyperalgesia induced by the incision as noted above due to inflammatory activation and also repetitive nociceptive barrages occurring as a result of the surgical repair; and (2) fentanyl produces rapid onset of hyperalgesic priming at both peripheral and central nociceptors as a result of prolongation of inflammatory-induced hyperalgesia.^94^ There was also a change in the fNIRS signal between nerve block and non-nerve block groups, which is one conundrum related to an analgesic effect. We expected the nerve block group to have less of a cortical response and also a decreased pain score in the early postoperative period versus the non-nerve block group (see Fig. 8). While we do not have a clear answer, there are a number of possibilities, including: (1) altered functional changes in interhemispheric communication: reports of alteration or distortion in responses in S1 may be a result of an increase in inhibitory interneuron activity for transcallosal pathways causing inappropriate functional responses in the ipsilateral hemisphere (see Ref. 96); (2) alterations in tactile inputs: the nerve block may have a differential effect on pain (C and Ad fibers) versus touch and proprioceptive inputs (AB fibers) (see Ref. 97). Given that the nerve block was administered to some patients while under anesthesia, there was no way to evaluate the efficacy during the anesthetic period.^98^ In line with this, sensory inputs may be present from other portions of the knee not involved in the nerve block’s targeted coverage; and (3) rewiring: neural blockade may cause rapid rewiring of S1 synaptic connections, as shown in prior fMRI studies in neuropathic pain patients,^99^ surgical disruption of nerves,^100^ or following local anesthetic blockade in noninjured animals.^101^^,^^102^ Local hyperexcitability of S1 and to frontal areas may result from rapid alteration of dendritic spines resulting from altered sensory input.^103^

### 4.4 Perioperative Pain Scores and Pain Algorithms

There are a few salient issues related to pain in this paper that deserve further investigation. The first is the ability to measure pain/nociceptive events as discussed above, and the second relates to the efficacy of the nerve block. Based on postsurgical self-reported VRS pain scores in the PACU (see Fig. 8), there are no differences in pain scores based on this small population (see caveats). In addition, there is no “blockade” of fNIRS activation with nerve block, as would be expected. As such, intraoperative monitoring of nociception, no matter what the anesthetic procedure is, would seem like a valuable asset in the surgical setting to provide information on inhibition of pain/nociception as measured by fNIRS. The third issue relates to the development of real-time measures: in addition to intraoperative notification of pain/nociceptive signals (and appropriate, timely analgesia or maintenance thereof), this approach may also provide a cumulative “pain load” that could be measured by applying algorithms to provide such a measure. Pain load may be a critical measure for predicting postoperative pain and, potentially, pain chronification.

The changes to hemoglobin concentrations as a response to pain can be measured in real time as a way to detect when the patient is feeling pain during surgery. From a series of repeatable results, an algorithm can be trained to receive previous time series values and foresee how the patient will respond to painful stimuli. Machine learning techniques practice on previous patient data where times of incision can be recorded.^104^ From here, the predictions can be made based on how patients with similar hemoglobin concentration levels have responded to pain.

### 4.5 Caveats

There are a number of caveats that need to be considered.

#### 4.5.1 Nature of the paradigm

Measures of nociception in a clinical setting do not allow for a paradigm that would allow for specific timing of anesthesia nor surgical interventions across all individuals. In our patients, measures are taken during routine procedures (i.e., no experimental constraints), this is consistent with our goal of developing a marker for uncontrolled nociception during surgery.

#### 4.5.2 Numbers of patients

We have measures in a total of 19 subjects, 11 with nerve blockade procedure. The use of repeated measures adds to the fidelity of the results. Although the number of patients was relatively small, for NB versus non-NB, our data still showed significant differences; clearly larger datasets would allow us to distinguish and other categories of differences, e.g., sex differences, age differences, etc.

#### 4.5.3 Efficacy of neural blockade

Based on our data, there was no difference between the postoperative pain scores between the two groups (see Fig. 8). This may indicate that the nerve blocks, although performed with ultrasound while patients are anesthetized, were not evaluated for efficacy. Furthermore, based on the innervation of the knee may require blockade of more than one block for complete blockade^105^ (note: in our group, the efficacy of ultrasound-guided NB in our patients was not evaluated postsurgery). However, local anesthetics may also exert an anti-inflammatory effect if the block performed (femoral nerve) is proximal to the surgery (knee). Other elements, such as nerve thickness, concentration and volume of the local anesthetic may affect the magnitude of the nerve block and outcome.^106^^,^^107^ Finally, there is evidence from clinical blocks that afferent activity still arrives at the central nervous system during local anesthesia in patients who are completely pain-free, that is, acutely, all sensation can be lost without blocking all impulses. The contribution of A-Beta nociceptive fibers in this situation is unknown.^108^

#### 4.5.4 Opioid analgesic effects on ${\mathrm{HbO}}_{2}$

Opioids may depress cortical activity^109^ as well as have an effect on hemoglobin oxygen saturation in volitional respiration as measured by fMRI.^110^^,^^111^ Under anesthesia, control of HbO2 may be obtained via inhalational control of oxygen.^112^ In our patient cohort, fentanyl was given at the beginning of the anesthetic procedure prior to any surgical intervention. Thus, the effects of fentanyl, even with higher plasma levels at the beginning of surgery, persist during the surgical intervention for at least 2 h (see above).

#### 4.5.5 Technical concerns

There are a number of technical concerns aside from ${\mathrm{HbO}}_{2}$ noted above. These include: (a) optimal maintenance of the cap during surgery—once the cap was fitted and the patients were under anesthesia, no adjustments were made, even if the head moved. This means that signal quality and/or regional specificity might have been compromised in some patients; (b) data quality in the somatosensory cortex can vary between subjects due to hair density, and in rare cases due to movement (although, the movement of patient is typically minimum after the surgery has commenced). Therefore, data were further processed using wavelet-based head motion correction and physiological noise regression to remove systemic noise sources; (c) correct correlation of observer time-locking events for later correlation with the fNIRS response (i.e., human error among research personnel involved in data collection); (d) methodological limitations of fNIRS equipment, such as limited spatial resolution (∼1 to 3 cm),^113^^,^^114^ and superficial penetration depth into the cortex, which limits investigation into deeper pain-related brain regions; (e) intraoperative evaluation under anesthesia makes it challenging to account for intersubject variability in analgesic state, and efficacy of peripheral nerve blockade; (f) distribution of activation over S1 observed in fNIRS—beyond “expected” somatotopy is expected as based on primate studies, there are multiple representations of the body in S1 within its various divisions and subdivisions (area 1, areas 2, 3a, and 3b) (see Ref. 115); (g) the period of time recorded when no procedures (painful and no painful) were being conducted (P0) could also be affected by the lingering effects of pain from previous incisions.

This was mitigated by taking the period of time furthest from any record of surgical procedures being conducted (these were chosen on a patient-by-patient basis). Alternatively, recording a minute from the preoperative data would ensure that the patient had not experience prior pain, but this would not be consistent with previous recordings, and would not account for the presence of nerve block in the 11 patients who received it. (g) Only incisions were considered “painful” when other procedures elicit minor responses which can build up over time. Future work can be made more sensitive to small pain events than this paper.

#### 4.5.6 Limitations of machine learning

While machine learning can train on existing patterns where pain does and does not occur, observing distinctions that clarify when a patient is undergoing a painful procedure without external interference, there are problems with these methods in their current incarnation. The current method pursued so far is based on time series analysis and the forecasting of future events. This is accomplished through looking at hemoglobin concentration levels in the two observed pain events P1 and P2 and comparing them with the control period P0. Typical time series models can incorporate known trends and cycles into the training cycle due to the large amount of accrued data; however, because of the nature of the experiments and the lack of a large sample size, these results are only based on the data that the 19 patients have been able to provide. Considerable work has gone into overcoming these deficiencies;^116^ however, while single subject training can lead to satisfactory results with consistent noise filtration and hemoglobin concentration normalization, at a group level these results can become difficult to train.

## 5 Conclusions

Surgery, by its very nature, produces tissue damage and inflammatory processes that result in an afferent barrage of nociceptor activity.^117^^,^^118^ Inhalational anesthesia only provides a state of unconsciousness but not analgesia, which is why it is frequently paired with the use of combined techniques including regional neural blockade and/or the use of opioids. However, the evaluation of pain/nociception during anesthesia lacks an objective marker that anesthesiologists can monitor and use to prevent inadequate analgesia. We propose using fNIRS responses/measures as potential markers of nociception during surgery. Our data suggest that such monitoring can be accomplished, and future work requires the development of algorithms and real-time monitoring to provide objective measures during surgical anesthesia.

## Code, Data, and Materials Availability

To request access to this dataset, please e-mail Stephen Green.

## References

[1] Agency for Healthcare Research and Quality (AHRQ), 2020, https://www.ahrq.gov (accessed 2020).

[2] Thapa S. P. Coaker G., “Genome sequences of two *Pseudomonas syringae* pv. tomato race 1 strains, isolated from tomato fields in California,” Genome Announc. 4(2), e01671 (2016). 10.1128/genomeA.01671-15 26966221 PMC4786671

[3] Rose J. et al., “Estimated need for surgery worldwide based on prevalence of diseases: a modelling strategy for the WHO Global Health Estimate,” Lancet Glob Health. 3(3 Suppl.), S13–S20 (2015). 10.1016/S2214-109X(15)70087-2 25926315 PMC5746187

[4] Brennan T. J. Vandermeulen E. P. Gebhart G. F., “Characterization of a rat model of incisional pain,” Pain 64(3), 493–502 (1996). 10.1016/0304-3959(95)01441-1 8783314

[5] Stubhaug A. et al., “Mapping of punctuate hyperalgesia around a surgical incision demonstrates that ketamine is a powerful suppressor of central sensitization to pain following surgery,” Acta Anaesthesiol Scand. 41(9), 1124–1132 (1997). 10.1111/j.1399-6576.1997.tb04854.x 9366932

[6] Vest S. A., “Prostatic malignancy,” Ciba Clin. Symp. 6(3), 93–103 (1954). 13161095

[7] Robleda G. et al., “Influence of preoperative emotional state on postoperative pain following orthopedic and trauma surgery,” Rev. Lat. Am. Enfermagem. 22(5), 785–791 (2014). 10.1590/0104-1169.0118.2481 25493674 PMC4292684

[8] Gan T. J., “Poorly controlled postoperative pain: prevalence, consequences, and prevention,” J Pain Res. 10, 2287–2298 (2017). 10.2147/JPR.S144066 29026331 PMC5626380

[9] Lindberg M. F. et al., “Symptoms, and psychological factors related to higher acute pain trajectories during hospitalization for total knee arthroplasty,” PLoS One 11(9), e0161681 (2016). POLNCL 1932-6203 10.1371/journal.pone.0161681 27583551 PMC5008744

[10] Borsook D. et al., “Surgically induced neuropathic pain: understanding the perioperative process,” Ann Surg. 257(3), 403–412 (2013). 10.1097/SLA.0b013e3182701a7b 23059501 PMC3546123

[11] Borsook D. et al., “When pain gets stuck: the evolution of pain chronification and treatment resistance,” Pain 159(12), 2421–2436 (2018). PAINDB 0304-3959 10.1097/j.pain.0000000000001401 30234696 PMC6240430

[12] Reddi D. Curran N., “Chronic pain after surgery: pathophysiology, risk factors and prevention,” Postgrad Med J. 90(1062), 222–227; quiz 226 (2014). 10.1136/postgradmedj-2013-132215 24572639

[r13] Althaus A. et al., “Development of a risk index for the prediction of chronic post-surgical pain,” Eur J Pain. 16(6), 901–910 (2012). 10.1002/j.1532-2149.2011.00090.x 22337572

[14] Kehlet H. Jensen T. S. Woolf C. J., “Persistent postsurgical pain: risk factors and prevention,” Lancet. 367(9522), 1618–1625 (2006). 10.1016/S0140-6736(06)68700-X 16698416

[15] Steegers M. A. et al., “Only half of the chronic pain after thoracic surgery shows a neuropathic component,” J Pain. 9(10), 955–961 (2008). 10.1016/j.jpain.2008.05.009 18632308

[16] Weibel S. et al., “Continuous intravenous perioperative lidocaine infusion for postoperative pain and recovery in adults,” Cochrane Database Syst Rev. 6, CD009642 (2018). 10.1002/14651858.CD009642.pub3 29864216 PMC6513586

[17] Morton D. L. Sandhu J. S. Jones A. K., “Brain imaging of pain: state of the art,” J. Pain Res. Volume 9, 613–624 (2016). 10.2147/JPR.S60433 PMC5019436 27660488

[18] Scholkmann F. et al., “A review on continuous wave functional near-infrared spectroscopy and imaging instrumentation and methodology,” Neuroimage 85(Pt. 1), 6–27 (2014). NEIMEF 1053-8119 10.1016/j.neuroimage.2013.05.004 23684868

[19] Villringer A. et al., “Near infrared spectroscopy (NIRS): a new tool to study hemodynamic changes during activation of brain function in human adults,” Neurosci. Lett. 154(1-2), 101–104 (1993). 10.1016/0304-3940(93)90181-J 8361619

[20] Aasted C. M. et al., “Frontal lobe hemodynamic responses to painful stimulation: a potential brain marker of nociception,” PLoS One 11(11), e0165226 (2016). POLNCL 1932-6203 10.1371/journal.pone.0165226 27806119 PMC5091745

[r21] Yucel M. A. et al., “Mayer waves reduce the accuracy of estimated hemodynamic response functions in functional near-infrared spectroscopy,” Biomed. Opt. Express. 7(8), 3078–3088 (2016). 10.1364/BOE.7.003078 27570699 PMC4986815

[r22] Kussman B. D. et al., “Capturing pain in the cortex during general anesthesia: near infrared spectroscopy measures in patients undergoing catheter ablation of arrhythmias,” PLoS One 11(7), e0158975 (2016). POLNCL 1932-6203 10.1371/journal.pone.0158975 27415436 PMC4944937

[r23] Yucel M. A. et al., “Short separation regression improves statistical significance and better localizes the hemodynamic response obtained by near-infrared spectroscopy for tasks with differing autonomic responses,” Neurophotonics 2(3), 035005 (2015). 10.1117/1.NPh.2.3.035005 26835480 PMC4717232

[r24] Becerra L. et al., “Brain measures of nociception using near-infrared spectroscopy in patients undergoing routine screening colonoscopy,” Pain 157(4), 840–848 (2016). 10.1097/j.pain.0000000000000446 26645550 PMC4794375

[r25] Aasted C. M. et al., “Anatomical guidance for functional near-infrared spectroscopy: AtlasViewer tutorial,” Neurophotonics 2(2), 020801 (2015). 10.1117/1.NPh.2.2.020801 26157991 PMC4478785

[r26] Yucel M. A. et al., “Specificity of hemodynamic brain responses to painful stimuli: a functional near-infrared spectroscopy study,” Sci. Rep. 5, 9469 (2015). 10.1038/srep09469 25820289 PMC4377554

[r27] Becerra L. et al., “Diffuse optical tomography activation in the somatosensory cortex: specific activation by painful vs. non-painful thermal stimuli,” PLoS One 4(11), e8016 (2009). POLNCL 1932-6203 10.1371/journal.pone.0008016 19956637 PMC2778627

[r28] Becerra L. et al., “Diffuse optical tomography of pain and tactile stimulation: activation in cortical sensory and emotional systems,” Neuroimage 41(2), 252–259 (2008). NEIMEF 1053-8119 10.1016/j.neuroimage.2008.01.047 18394924 PMC2728450

[r29] Peng K. et al., “Brodmann area 10: collating, integrating and high level processing of nociception and pain,” Prog. Neurobiol. 161, 1–22 (2018). 10.1016/j.pneurobio.2017.11.004 29199137 PMC5826795

[30] Peng K. et al., “Using prerecorded hemodynamic response functions in detecting prefrontal pain response: a functional near-infrared spectroscopy study,” Neurophotonics 5(1), 011018 (2018). 10.1117/1.NPh.5.1.011018 29057285 PMC5641587

[31] Peng K. et al., “Morphine attenuates fNIRS signal associated with painful stimuli in the medial frontopolar cortex (medial BA 10),” Front. Hum. Neurosci. 12, 394 (2018). 10.3389/fnhum.2018.00394 30349466 PMC6186992

[32] Karunakaran K. D. et al., “NIRS measures in pain and analgesia: fundamentals, features, and function,” Neurosci. Biobehav. Rev. 120, 335 (2020). 10.1016/j.neubiorev.2020.10.023 33159918

[33] Tang C. Y. Ramani R., “fMRI and anesthesia,” Int. Anesthesiol. Clin. 54(1), 129–142 (2016). 10.1097/AIA.0000000000000081 26655513

[r34] Bonhomme V. et al., “Neural correlates of consciousness during general anesthesia using functional magnetic resonance imaging (fMRI),” Arch. Ital. Biol. 150(2-3), 155–163 (2012). 10.4449/aib.v150i2.1242 23165875

[35] Antognini J. F et al., “Isoflurane anesthesia blunts cerebral responses to noxious and innocuous stimuli: a fMRI study,” Life Sci. 61(24), PL349–PL354 (1997). 10.1016/S0024-3205(97)00960-0 9399635

[36] Kim W. Kim S. K. Nabekura J., “Functional and structural plasticity in the primary somatosensory cortex associated with chronic pain,” J. Neurochem. 141(4), 499–506 (2017). 10.1111/jnc.14012 28278355

[r37] Vierck C. J. et al., “Role of primary somatosensory cortex in the coding of pain,” Pain 154(3), 334–344 (2013). 10.1016/j.pain.2012.10.021 23245864 PMC4501501

[38] Willis W. D. Jr., “Pain pathways in the primate,” Prog. Clin. Biol. Res. 176, 117–133 (1985). 3923492

[39] Duerden E. G. Albanese M. C., “Localization of pain-related brain activation: a meta-analysis of neuroimaging data,” Hum. Brain. Mapp. 34(1), 109–149 (2013). 10.1002/hbm.21416 22131304 PMC6869965

[40] Eroglu A. et al., “Regional anesthesia for postoperative pain control,” Biomed. Res. Int. 2014, 309606 (2014). 10.1155/2014/309606 25054137 PMC4087300

[41] Levesque D. Di Paolo T., “Rapid conversion of high into low striatal D2-dopamine receptor agonist binding states after an acute physiological dose of 17 beta-estradiol,” Neurosci. Lett. 88(1), 113–118 (1988). 10.1016/0304-3940(88)90324-2 2969467

[42] Lee H. J. Yeomans D. C., “Opioid induced hyperalgesia in anesthetic settings,” Korean J. Anesthesiol. 67(5), 299–304 (2014). 10.4097/kjae.2014.67.5.299 25473457 PMC4252340

[43] Kim S. H. et al., “Intraoperative use of remifentanil and opioid induced hyperalgesia/acute opioid tolerance: systematic review,” Front. Pharmacol. 5, 108 (2014). 10.3389/fphar.2014.00108 24847273 PMC4021143

[44] Mall N. A. et al., “Incidence and trends of anterior cruciate ligament reconstruction in the United States,” Am. J. Sports Med. 42(10), 2363–2370 (2014). 10.1177/0363546514542796 25086064

[45] Nader A. et al., “Single-dose adductor canal block with local infiltrative analgesia compared with local infiltrate analgesia after total knee arthroplasty: a randomized, double-blind, placebo-controlled trial,” Reg. Anesth. Pain Med. 41(6), 678–684 (2016). 10.1097/AAP.0000000000000494 27776098

[46] Sawhney M. et al., “Pain after unilateral total knee arthroplasty: a prospective randomized controlled trial examining the analgesic effectiveness of a combined adductor canal peripheral nerve block with periarticular infiltration versus adductor canal nerve block alone versus periarticular infiltration alone,” Anesth. Analg. 122(6), 2040–2046 (2016). 10.1213/ANE.0000000000001210 27028771

[47] Ramlogan R. Tierney S. McCartney C. J. L., “Anterior cruciate ligament repair and peripheral nerve blocks: time to change our practice?” Br. J. Anaesth. 123(2), e186–e188 (2019). 10.1016/j.bja.2019.05.028 31202563 PMC6676314

[48] Carvalho L. H. Jr. et al., “Reducing the length of hospital stay after total knee arthroplasty: influence of femoral and sciatic nerve block,” Rev. Assoc. Med. Bras. 61(1), 40–43 (2015). 10.1590/1806-9282.61.01.040 25909207

[49] Raja S. N. et al., “The revised international association for the study of pain definition of pain: concepts, challenges, and compromises,” Pain 161(9), 1976–1982 (2020). 10.1097/j.pain.0000000000001939 32694387 PMC7680716

[50] Zhao F. et al., “Qualification of fMRI as a biomarker for pain in anesthetized rats by comparison with behavioral response in conscious rats,” Neuroimage 84, 724–732 (2014). NEIMEF 1053-8119 10.1016/j.neuroimage.2013.09.036 24064074

[r51] Zhao F. et al., “fMRI of pain processing in the brain: a within-animal comparative study of BOLD vs. CBV and noxious electrical vs. noxious mechanical stimulation in rat,” Neuroimage 59(2), 1168–1179 (2012). NEIMEF 1053-8119 10.1016/j.neuroimage.2011.08.002 21856430

[52] Borsook D. Becerra L., “CNS animal fMRI in pain and analgesia,” Neurosci. Biobehav. Rev. 35(5), 1125–1143 (2011). 10.1016/j.neubiorev.2010.11.005 21126534 PMC3076623

[53] Constant I. Sabourdin N., “Monitoring depth of anesthesia: from consciousness to nociception. A window on subcortical brain activity,” Paediatr. Anaesth. 25(1), 73–82 (2015). 10.1111/pan.12586 25410376

[r54] Grunwald J. E. et al., “Laser Doppler velocimetry study of retinal circulation in diabetes mellitus,” Arch. Ophthalmol. 104(7), 991–996 (1986). 10.1001/archopht.1986.01050190049038 2942132

[55] Untergehrer G. et al., “Effects of propofol, sevoflurane, remifentanil, and (S)-ketamine in subanesthetic concentrations on visceral and somatosensory pain-evoked potentials,” Anesthesiology 118(2), 308–317 (2013). ANESAV 0003-3022 10.1097/ALN.0b013e318279fb21 23254146

[56] Rodriguez-Merchan E. C., “Primary repair of the anterior cruciate ligament: a review of recent literature (2016-2017),” Arch. Bone Jt. Surg. 7(3), 297–300 (2019). 31312690 PMC6578482

[57] Paschos N. K. Howell S. M., “Anterior cruciate ligament reconstruction: principles of treatment,” EFORT Open Rev. 1(11), 398–408 (2016). 10.1302/2058-5241.1.160032 28461919 PMC5367541

[58] Brigadoi S. Cooper R. J., “How short is short? Optimum source-detector distance for short-separation channels in functional near-infrared spectroscopy,” Neurophotonics 2(2), 025005 (2015). 10.1117/1.NPh.2.2.025005 26158009 PMC4478880

[59] StockTrek Medical, Anatomy of Human Knee Joint, Poster, Zazzle.

[60] Molavi B. Dumont G. A., “Wavelet-based motion artifact removal for functional near-infrared spectroscopy,” Physiol. Meas. 33(2), 259–270 (2012). 10.1088/0967-3334/33/2/259 22273765

[61] Pfeifer M. D. Scholkmann F. Labruyere R., “Signal processing in functional near-infrared spectroscopy (fNIRS): methodological differences lead to different statistical results,” Front. Hum. Neurosci. 11, 641 (2018). 10.3389/fnhum.2017.00641 29358912 PMC5766679

[r62] Kamran M. A. Mannan M. M. Jeong M. Y., “Cortical signal analysis and advances in functional near-infrared spectroscopy signal: a review,” Front. Hum. Neurosci. 10, 261 (2016). 10.3389/fnhum.2016.00261 27375458 PMC4899446

[63] Huppert T. J. et al., “HomER: a review of time-series analysis methods for near-infrared spectroscopy of the brain,” Appl. Opt. 48(10), D280–298 (2009). 10.1364/AO.48.00D280 19340120 PMC2761652

[64] Sawilowsky S. S., “New effect size rules of thumb,” J. Mod. Appl. Stat. Methods 8(2), 597–599 (2009). 10.22237/jmasm/1257035100

[r65] Cohen J., Statistical Power Analysis for the Behavioral Sciences, 2nd ed., Lawrence Erlbaum Associates, Hillsdale, New Jersey (1988).

[66] Koo T. K. Li M. Y., “A guideline of selecting and reporting intraclass correlation coefficients for reliability research,” J. Chiropr. Med. 15(2), 155–163 (2016). 10.1016/j.jcm.2016.02.012 27330520 PMC4913118

[67] Lichtner G. et al., “Nociceptive activation in spinal cord and brain persists during deep general anaesthesia,” Br. J. Anaesth. 121(1), 291–302 (2018). 10.1016/j.bja.2018.03.031 29935584

[68] Lichtner G. et al., “Effects of propofol anesthesia on the processing of noxious stimuli in the spinal cord and the brain,” Neuroimage 172, 642–653 (2018). NEIMEF 1053-8119 10.1016/j.neuroimage.2018.02.003 29421324

[69] Torricelli A. et al., “Time domain functional NIRS imaging for human brain mapping,” Neuroimage 85(Pt. 1), 28–50 (2014). NEIMEF 1053-8119 10.1016/j.neuroimage.2013.05.106 23747285

[70] “IASP announces revised definition of pain,” 2020, https://www.iasp-pain.org/PublicationsNews/NewsDetail.aspx?ItemNumber=10475 (accessed 2020).

[71] Dubin A. E. Patapoutian A., “Nociceptors: the sensors of the pain pathway,” J. Clin. Invest. 120(11), 3760–3772 (2010). 10.1172/JCI42843 21041958 PMC2964977

[72] Amaya F. et al., “Tissue injury and related mediators of pain exacerbation,” Curr. Neuropharmacol. 11(6), 592–597 (2013). 10.2174/1570159X11311060003 24396335 PMC3849785

[73] Latremoliere A. Woolf C. J., “Central sensitization: a generator of pain hypersensitivity by central neural plasticity,” J. Pain 10(9), 895–926 (2009). 10.1016/j.jpain.2009.06.012 19712899 PMC2750819

[74] Woolf C. J. Chong M. S., “Preemptive analgesia--treating postoperative pain by preventing the establishment of central sensitization,” Anesth. Analg. 77(2), 362–379 (1993). 10.1213/00000539-199377020-00026 8346839

[75] Ji R. R. et al., “Neuroinflammation and central sensitization in chronic and widespread pain,” Anesthesiology 129(2), 343–366 (2018). ANESAV 0003-3022 10.1097/ALN.0000000000002130 29462012 PMC6051899

[76] Theuvenet P. J. et al., “Anesthetic block of pain-related cortical activity in patients with peripheral nerve injury measured by magnetoencephalography,” Anesthesiology 115(2), 375–386 (2011). ANESAV 0003-3022 10.1097/ALN.0b013e31821f6562 21685789

[77] Lahti K. M. et al., “Comparison of evoked cortical activity in conscious and propofol-anesthetized rats using functional MRI,” Magn. Reson. Med. 41(2), 412–416 (1999). 10.1002/(SICI)1522-2594(199902)41:2<412::AID-MRM28>3.0.CO;2-3 10080292

[78] Ririe D. G. et al., “Incisional nociceptive input impairs attention-related behavior and is associated with reduced neuronal activity in the prefrontal cortex in rats,” Anesthesiology 129(4), 778–790 (2018). ANESAV 0003-3022 10.1097/ALN.0000000000002325 29952818 PMC6419730

[79] Apkarian A. V. et al., “Human brain mechanisms of pain perception and regulation in health and disease,” Eur. J. Pain 9(4), 463 (2005). 10.1016/j.ejpain.2004.11.001 15979027

[80] Nakamura A. et al., “Somatosensory homunculus as drawn by MEG,” Neuroimage 7(4 Pt. 1), 377–386 (1998). NEIMEF 1053-8119 10.1006/nimg.1998.0332 9626677

[81] Yang T. T. et al., “Noninvasive somatosensory homunculus mapping in humans by using a large-array biomagnetometer,” Proc. Natl. Acad. Sci. U. S. A. 90(7), 3098–3102 (1993). 10.1073/pnas.90.7.3098 8464929 PMC46244

[82] Calford M. B., “Mechanisms for acute changes in sensory maps,” Adv. Exp. Med. Biol. 508, 451–460 (2002). 10.1007/978-1-4615-0713-0_51 12171142

[83] Buchner H. Kauert C. Radermacher I., “Short-term changes of finger representation at the somatosensory cortex in humans,” Neurosci. Lett. 198(1), 57–59 (1995). 10.1016/0304-3940(95)11950-2 8570097

[84] Rossini P. M. et al., “Short-term brain ‘plasticity’ in humans: transient finger representation changes in sensory cortex somatotopy following ischemic anesthesia,” Brain Res. 642(1-2), 169–177 (1994). BRREAP 0006-8993 10.1016/0006-8993(94)90919-9 8032877

[85] Ong W. Y. Stohler C. S. Herr D. R., “Role of the prefrontal cortex in pain processing,” Mol. Neurobiol. 56(2), 1137–1166 (2019). 10.1007/s12035-018-1130-9 29876878 PMC6400876

[86] Seminowicz D. A. Moayedi M., “The dorsolateral prefrontal cortex in acute and chronic pain,” J. Pain 18(9), 1027–1035 (2017). 10.1016/j.jpain.2017.03.008 28400293 PMC5581265

[87] Kucyi A. Davis K. D., “The dynamic pain connectome,” Trends Neurosci. 38(2), 86–95 (2015). TNSCDR 0166-2236 10.1016/j.tins.2014.11.006 25541287

[88] Ziesenitz V. C. et al., “Pharmacokinetics of fentanyl and its derivatives in children: a comprehensive review,” Clin. Pharmacokinet. 57(2), 125–149 (2018). 10.1007/s40262-017-0569-6 28688027 PMC5756700

[89] Umino A. et al., “Evidence for tonic control by the GABAA receptor of extracellular D-serine concentrations in the medial prefrontal cortex of rodents,” Front. Mol. Neurosci. 10, 240 (2017). 10.3389/fnmol.2017.00240 28824371 PMC5539225

[90] Zhang Y. et al., “Inhaled anesthetics have hyperalgesic effects at 0.1 minimum alveolar anesthetic concentration,” Anesth. Analg. 91(2), 462–466 (2000). 10.1097/00000539-200008000-00044 10910869

[91] “Fentanyl citrate injection, USP,” US Food and Drug Administration (FDA).

[92] Adler L. J. et al., “Regional brain activity changes associated with fentanyl analgesia elucidated by positron emission tomography,” Anesth. Analg. 84(1), 120–126 (1997). 10.1213/00000539-199701000-00023 8989012

[93] Wagner K. J. et al., “Imaging human cerebral pain modulation by dose-dependent opioid analgesia: a positron emission tomography activation study using remifentanil,” Anesthesiology 106(3), 548–556 (2007). ANESAV 0003-3022 10.1097/00000542-200703000-00020 17325514

[94] Peng Y. Z. Li X. X. Wang Y. W., “Effects of parecoxib and fentanyl on nociception-induced cortical activity,” Mol. Pain. 6, 1744-8069-6-3 (2010). 10.1186/1744-8069-6-3 PMC2819047 20089200

[95] Kim S. K. Eto K. Nabekura J., “Synaptic structure and function in the mouse somatosensory cortex during chronic pain: *in vivo* two-photon imaging,” Neural Plast. 2012, 640259 (2012). 10.1155/2012/640259 22530157 PMC3317022

[96] Araldi D. et al., “Fentanyl induces rapid onset hyperalgesic priming: type I at peripheral and type II at central nociceptor terminals,” J. Neurosci. 38(9), 2226–2245 (2018). 10.1523/JNEUROSCI.3476-17.2018 29431655 PMC5830512

[97] Calford M. B. Tweedale R., “Interhemispheric transfer of plasticity in the cerebral cortex,” Science 249(4970), 805–807 (1990). SCIEAS 0036-8075 10.1126/science.2389146 2389146

[98] Li N. et al., “Optogenetic-guided cortical plasticity after nerve injury,” Proc. Natl. Acad. Sci. U. S. A. 108(21), 8838–8843 (2011). 10.1073/pnas.1100815108 21555573 PMC3102379

[99] Paqueron X. et al., “Time sequence of sensory changes after upper extremity block: swelling sensation is an early and accurate predictor of success,” Anesthesiology 101(1), 162–168 (2004). ANESAV 0003-3022 10.1097/00000542-200407000-00025 15220786

[100] Karl A. et al., “Reorganization of motor and somatosensory cortex in upper extremity amputees with phantom limb pain,” J. Neurosci. 21(10), 3609–3618 (2001). 10.1523/JNEUROSCI.21-10-03609.2001 11331390 PMC6762494

[101] Merzenich M. M. et al., “Topographic reorganization of somatosensory cortical areas 3b and 1 in adult monkeys following restricted deafferentation,” Neuroscience 8(1), 33–55 (1983). 10.1016/0306-4522(83)90024-6 6835522

[102] Faggin B. M. Nguyen K. T. Nicolelis M. A., “Immediate and simultaneous sensory reorganization at cortical and subcortical levels of the somatosensory system,” Proc. Natl. Acad. Sci. U. S. A. 94(17), 9428–9433 (1997). 10.1073/pnas.94.17.9428 9256499 PMC23207

[103] Doetsch G. S. et al., “Short-term plasticity in primary somatosensory cortex of the rat: rapid changes in magnitudes and latencies of neuronal responses following digit denervation,” Exp. Brain Res. 112(3), 505–512 (1996). 10.1007/BF00227956 9007552

[104] Boonstra A. M. et al., “Cut-off points for mild, moderate, and severe pain on the numeric rating scale for pain in patients with chronic musculoskeletal pain: variability and influence of sex and catastrophizing,” Front. Psychol. 7, 1466 (2016). 10.3389/fpsyg.2016.01466 27746750 PMC5043012

[105] Ahmed N. K. et al., “An empirical comparison of machine learning models for time series forecasting,” Econom. Rev. 29(5-6), 594–621 (2010). ECREEP 10.1080/07474938.2010.481556

[106] Kukreja P. et al., “A summary of the anatomy and current regional anesthesia practices for postoperative pain management in total knee arthroplasty,” Cureus 10(6), e2755 (2018). 10.7759/cureus.2755 30094112 PMC6080732

[107] Pushpanathan E. et al., “A systematic review of postoperative pain outcome measurements utilised in regional anesthesia randomized controlled trials,” Anesthesiol Res. Pract. 2018, 9050239 (2018). 10.1155/2018/9050239 30151005 PMC6087609

[108] Vadhanan P. Tripaty D. K. Adinarayanan S., “Physiological and pharmacologic aspects of peripheral nerve blocks,” J. Anaesthesiol. Clin. Pharmacol. 31(3), 384–393 (2015). 10.4103/0970-9185.161679 26330722 PMC4541190

[109] Djouhri L. Lawson S. N., “Abeta-fiber nociceptive primary afferent neurons: a review of incidence and properties in relation to other afferent A-fiber neurons in mammals,” Brain Res. Brain Res. Rev. 46(2), 131–145 (2004). 10.1016/j.brainresrev.2004.07.015 15464202

[110] Pattinson K. T. et al., “Opioids depress cortical centers responsible for the volitional control of respiration,” J. Neurosci. 29(25), 8177–8186 (2009). 10.1523/JNEUROSCI.1375-09.2009 19553457 PMC6666048

[111] Pattinson K. T. Wise R. G., “Imaging the respiratory effects of opioids in the human brain,” Adv. Exp. Med. Biol. 903, 145–156 (2016). 10.1007/978-1-4899-7678-9_10 27343094

[112] Ottolenghi S. et al., “Hyperoxia and oxidative stress in anesthesia and critical care medicine,” Minerva Anestesiol. 86(1), 64–75 (2020). MIANAP 10.23736/S0375-9393.19.13906-5 31680497

[113] Shoaib Z. et al., “Approach to optimize 3-dimensional brain functional activation image with high resolution: a study on functional near-infrared spectroscopy,” Biomed. Opt. Express 10(9), 4684–4710 (2019). 10.1364/BOE.10.004684 31565519 PMC6757466

[114] Yucel M. A. et al., “Functional near infrared spectroscopy: enabling routine functional brain imaging,” Curr. Opin. Biomed. Eng. 4, 78–86 (2017). 10.1016/j.cobme.2017.09.011 29457144 PMC5810962

[115] Kaas J. H. et al., “Multiple representations of the body within the primary somatosensory cortex of primates,” Science 204(4392), 521–523 (1979). SCIEAS 0036-8075 10.1126/science.107591 107591

[116] Masini R. P. Medeiros M. C. Mendes E.F., “Machine learning advances for time series forecasting,” arXiv:2012.12802 (2020).

[117] Xu J. Brennan T. J., “Guarding pain and spontaneous activity of nociceptors after skin versus skin plus deep tissue incision,” Anesthesiology 112(1), 153–164 (2010). ANESAV 0003-3022 10.1097/ALN.0b013e3181c2952e 19996955 PMC2907154

[118] Xu J. Brennan T. J., “Comparison of skin incision vs. skin plus deep tissue incision on ongoing pain and spontaneous activity in dorsal horn neurons,” Pain 144(3), 329–339 (2009). PAINDB 0304-3959 10.1016/j.pain.2009.05.019 19527922 PMC2759309

