# Supplementary material for: fNIRS brain measures of ongoing nociception during surgical incisions under anesthesia
Source: Neurophotonics. 2022 Jan 27;9(1):015002. doi: 10.1117/1.NPh.9.1.015002 (PMC8794294; doi:10.1117/1.NPh.9.1.015002)
Supplement: Supplementary file 1 [file NPh_009_015002_SD001.pdf]

Supplementary Material

Patient	Weight (kg)	Midazolam (mg)	Fentanyl (mcg)	Sufentanil (mcg)	Propofol (mg)	Propofol infusion (mg)	Volatile Agent
1	52.8	2	100		150	288	S
2	81	2		50	200	-	S
3	55	2	100		200	-	S
4	55.5	2	100		200	235	S
5	38.8	2	100		200	-	S
6	71.5	2	100		200	200	S
7	92.4	2	250		200	-	S
8	84.7	2	250		0[y]	390	S, D
9	63.2	2	100		150	224	Iso
10	56	2	100		200	354	S
11	66.8	2			250	-	S
12	77.4	2	150		200	480	S
13	87.8	2	250		Unknown	841	S
14	52.5	2	150		170	158	S
15	70.8	2	150		200	372	S
16	82	2	100		300	951	S
17	65.3	2	100		200	372	S, D
18	68.4	2	100		150	-	S
19	70.2	2	100		200	-	S

Table S1a: Anesthetic Technique: General Anesthesia Abbreviations: S, sevoflurane; D, desflurane; I, isoflurane; Epi, epinephrine; R, right; L, left; ss, single shot; c, catheter. [y]Sevoflurane induction.

Patient	Regional Anesthesia	Ropivacaine Dose	Time - Block Placement to Incision (mins.)	Time of block placement	Time of incision
1	R Adductor Canal (ss)	20mL 0.2%	16	8:10	8:26
2	-	-			
3	-	-			
4	R Adductor Canal (ss)	15mL 0.2%	18	8:43	9:01
5	L Adductor Canal (ss) + L Lateral Femoral Cutaneous Nerve (ss)	10mL 0.2% + 20mcg clonidine x 2	21	7:49	8:10
6	-	-			
7	R Adductor Canal (ss)	30mL 0.2% + 100mcg dexmedetomidine	25	12:31	12:56
8	L Adductor Canal (ss)	30mL 0.35%	27	14:48	15:15
9	R Adductor Canal (ss)	30mL 0.35% + 20mcg clonidine	14	13:44	13:58
10	R Adductor Canal (ss)	20mL 0.2%	14	11:00	11:14
11	-				
12	L Adductor Canal (ss)	17mL 0.35%	15	9:55	10:10
13	L Adductor Canal (ss)	20mL 0.2%	18	7:50	8:08
14	-	-			
15	-	-			
16	L Adductor Canal (c)	20mL 0.35%	37	12:48	13:25
17	L Adductor Canal (ss)	18 mL 0.35%	53*	10:30	11:23
18	-	-			
19	-	-	12	12:01	12:13

Table S1b: Anesthetic Technique: Regional Blockade *Nerve block placed prior to induction of anesthesia.

Patient	Local Anesthetic Infiltration by Surgeon (0.25% Bupivacaine with epi. 1:200,000) and Timing	Duration of Surgery (Incision-End) (mins.)	Acetaminophen (mg)	Morphine (mg)	Hydromorphone (mg)
1	26 mL Incision	111	650	1	
2	30 mL End	82	650		0.6
3	27.6 mL End	46	650		1.4
4	-	75	650		0.5
5	15 mL Incision	29	-		0.6
6	30 mL End	49	650		0.4
7	30 mL Incision	106	650		0.8
8	30 mL End	105	650		
9	10 mL End	63	650		0.6
10	28 mL Incision	80	650		
11	30 mL End	70	650		1
12	30 mL Incision	73	650		0.4
13	30 mL Incision	117	650		0.6
14	-	36	650		
15	30 mL End	48	650		0.3
16	20 mL Incision	122	650		1
17	20 mL Incision	128	650		1.6
18	30 mL End	116	650		0.6
19	30 mL Incision	125	650		0.8

Table S1c: Anesthetic Technique: Local Anesthetics and Analgesics

Patient	Ketorolac (mg)	Diazepam (mg)	Dexmedetomidine (mcg)	Ondansetron (mcg)	Dexamethasone (mcg)	Scopolamine (mcg)
1	30	-	-	4	-	-
2	-	5	-	4	4	-
3	-	-	-	4	-	-
4	18	5	-	4	4	-
5	30	-	-	4	4	-
6	30	-	16	4	8	1.5
7	-	-		4	4	-
8	-	5	57.17 (infusion)	4	4	-
9	30	5	-	4	4	-
10	27	-	-	4	4	-
11	-	-	-	4	6	-
12	30	2.5	8	4	4	-
13	-	2.5	12	4	8	1.5
14	-	-	12	4	4	-
15	30	-	8	4	6	-
16	30	5	-	4	-	-
17	-	7.5	-	4	4	-
18	-	5	-	4	4	-
19	30	2.5	-	4	4	-

Table S1d: Anesthetic Technique: Post-Surgical Treatment

P-Values	Lateral FC	Lateral PreFC	Medial PreFC	Superior S1	Central S1	Inferior S1
P0 Oxy CL	0.1342 (3)	0.9607 (3)	0.3100 (4)	0.2224 (5)	0.1183 (6)	0.0783 (5)
P1 Oxy CL	0.3104 (4)	0.4841 (2)	0.1789 (2)	**0.0276 (5)**	**0.0052 (4)***	0.0580 (5)
P2 Oxy CL	0.1614 (4)	0.8135 (2)	0.8436 (3)	0.1183 (4)	0.1883 (4)	0.0816 (5)
P0 Deoxy CL	0.2817 (3)	0.3367 (4↑)	0.1652 (4)	0.8422 (3)	**0.0091 (6)**	0.1379 (4)
P1 Deoxy CL	0.3964 (3)	0.1831 (3)	0.2615 (3↓)	0.5007 (3)	**0.0075 (6)***	0.1583 (3)
P2 Deoxy CL	0.4809 (4)	0.6213 (3)	0.4304 (4)	0.5876 (2)	**0.0088 (6)**	0.3695 (4)
P0 Total CL	**0.0318 (5)**	0.8702 (3)	0.5260 (3↓)	0.4349 (5)	0.0940 (5)	**0.0079 (5)**
P1 Total CL	0.0785 (5)	0.5152 (4)	0.5263 (3)	**0.0299** (3)	0.0679 (4)	**0.0353 (5)**
P2 Total CL	0.0725 (5)	0.6125 (2)	0.3434 (3)	0.2974 (3)	0.2641 (5)	0.0796 (6)
P0 Oxy IL	0.4519 (3)	0.6385 (2)	0.2753 (5)	0.4156 (5)	0.1767 (4)	0.6442 (2)
P1 Oxy IL	0.4292 (2)	0.8053 (3)	0.1772 (3)	0.1034 (3)	0.2033 (5)	0.9480 (3)
P2 Oxy IL	0.9331 (3)	0.6244 (3)	0.4367 (4)	0.4814 (3↓)	**0.0384 (4)**	0.4996 (3)
P0 Deoxy IL	0.1520 (4)	0.3377 (3)	0.2175 (4)	0.7604 (2↓)	0.2304 (4)	0.4446 (2)
P1 Deoxy IL	0.2828 (4)	0.5380 (3)	0.2108 (4↑)	0.7326 (3↓↓)	0.1262 (5)	0.7464 (5↓)
P2 Deoxy IL	0.2081 (4)*	0.5219 (3)	0.2494 (4)	0.4791 (2↓↓)	0.0756 (4)	0.0810 (3)
P0 Total IL	0.8335 (2)	0.6281 (2)	0.1872 (4)	0.7855 (3)	0.3057 (2)	0.4638 (2)
P1 Total IL	0.9285 (3)	0.9350 (3)	0.1242 (3)	0.5444 (3)	0.7073 (3↓)	0.8267 (2)
P2 Total IL	0.3326 (2)	0.5866 (3)	0.3114 (4)	0.6588 (2)	0.4170 (3)	0.9026 (3)

Table S2: Nerve Block Effects, Effect Size and Intra Class Correlation in Cortices. p-value for the two-sample t-test between NB and non-NB groups for HbO concentrations. $p<0.05$ (shown in **bold**) that there is a significant effect between the application of NB and the area under the standardized curves (*italicized* areas are also significant after FDR at $\alpha=0.05$). The Cohen's D Formula shows the effect size of each relationship. The sign shows direction of effect from NB to non-NB while effect size is shown to be either very small (1), small (2), medium (3), large (4), very large (5) or huge (6) with absolute values between 0 and 2.[106,107] The Intra-Class Correlation (ICC) describes the strength of resembles between units in each ROI. Koo and Li (2016)[108] describe ICC<0.5 as poor, 0.5<ICC<0.75 as moderate (marked with one arrow), 0.75<ICC<0.90 as good (marked with two arrows) and 0.9<ICC as excellent (marked with three arrows). Arrows point upwards (↑) for NB groups and downwards (↓) for non-NB groups, with notable ICC differences between pain and no pain underlined.

p-Values	PreFC	S1	PreFC/S1	Total
Time Intercept: P0 Oxy CL	0.1120	0.4551	0.0808	0.2357
Time Intercept: P1 Oxy CL	0.6517	0.1088	0.1771	0.2892
Time Intercept: P2 Oxy CL	0.1329	0.0839	***0.0011****	0.0644
Nerve Block: P0 Oxy CL	0.0922	0.1999	0.0667	0.2515
Nerve Block: P1 Oxy CL	0.7500	0.2042	***0.0320***	***0.0417***
Nerve Block: P2 Oxy CL	0.5626	0.1997	***0.0077****	0.0761
Time Intercept: P0 Deoxy CL	0.3992	0.2252	0.1748	0.4553
Time Intercept: P1 Deoxy CL	0.4438	0.1664	0.0988	0.4425
Time Intercept: P2 Deoxy CL	0.1587	0.4111	*0.0203*	*0.0442*
Nerve Block: P0 Deoxy CL	0.2872	0.2854	0.5126	0.6209
Nerve Block: P1 Deoxy CL	0.0655	0.3466	0.0988	*0.0295*
Nerve Block: P2 Deoxy CL	0.3404	0.5348	0.1653	0.3772
Time Intercept: P0 Total CL	0.1800	0.5826	0.0674	0.1984
Time Intercept: P1 Total CL	0.1418	0.0977	0.2151	0.1730
Time Intercept: P2 Total CL	0.2554	0.2013	*0.0175*	0.1069
Nerve Block: P0 Total CL	0.3198	0.1554	0.3651	0.4222
Nerve Block: P1 Total CL	0.7075	0.1786	*0.0211*	0.0986
Nerve Block: P2 Total CL	0.2954	0.6943	*0.0210*	0.1859
Time Intercept: P0 Oxy IL	0.5391	0.4678	0.0972	0.2793
Time Intercept: P1 Oxy IL	0.5389	0.1719	0.1205	0.1714
Time Intercept: P2 Oxy IL	0.2626	0.1069	***0.0103***	*0.0199*
Nerve Block: P0 Oxy IL	0.2279	0.1695	0.1291	0.3816
Nerve Block: P1 Oxy IL	0.1931	0.2478	***0.0228****	***0.0280****
Nerve Block: P2 Oxy IL	0.1450	0.3676	***0.0451****	0.0186
Time Intercept: P0 Deoxy IL	0.1546	0.5963	0.1360	0.0956
Time Intercept: P1 Deoxy IL	0.2453	0.2540	0.5608	0.3080
Time Intercept: P2 Deoxy IL	0.3827	0.1375	0.3145	0.2388
Nerve Block: P0 Deoxy IL	0.2508	0.2089	0.1832	0.1505
Nerve Block: P1 Deoxy IL	0.2343	0.2153	0.1373	0.2268
Nerve Block: P2 Deoxy IL	0.2778	0.1747	0.2999	0.3758
Time Intercept: P0 Total IL	0.0757	0.2518	*0.0094*	0.5448
Time Intercept: P1 Total IL	0.2554	0.4136	0.1258	0.2786
Time Intercept: P2 Total IL	0.1948	0.1013	*0.0332*	0.0992
Nerve Block: P0 Total IL	0.2407	0.4112	*0.0044*	0.1646
Nerve Block: P1 Total IL	0.3218	0.5336	*0.0185*	0.1009
Nerve Block: P2 Total IL	0.1031	0.0897	0.0945	0.0596

Table S3: Significant fNIRS changes. *p*-values of the effect of Time and Nerve Block on correlations for Δ[HbO]. $p<0.05$ are highlighted in **bold** and show a significant effect between the concentration and the respective component for first and last pain event, along with the control correlations for P0, P1 and P2 on the contralateral (CL) and ipsilateral cortices (IL) respective to the knee that was operated on. *p*-values are also statistically significant after FDR where α=0.05 (See **Fig. 5**) are marked with an asterisk (*). Notable patterns during pain events are *italicized*.

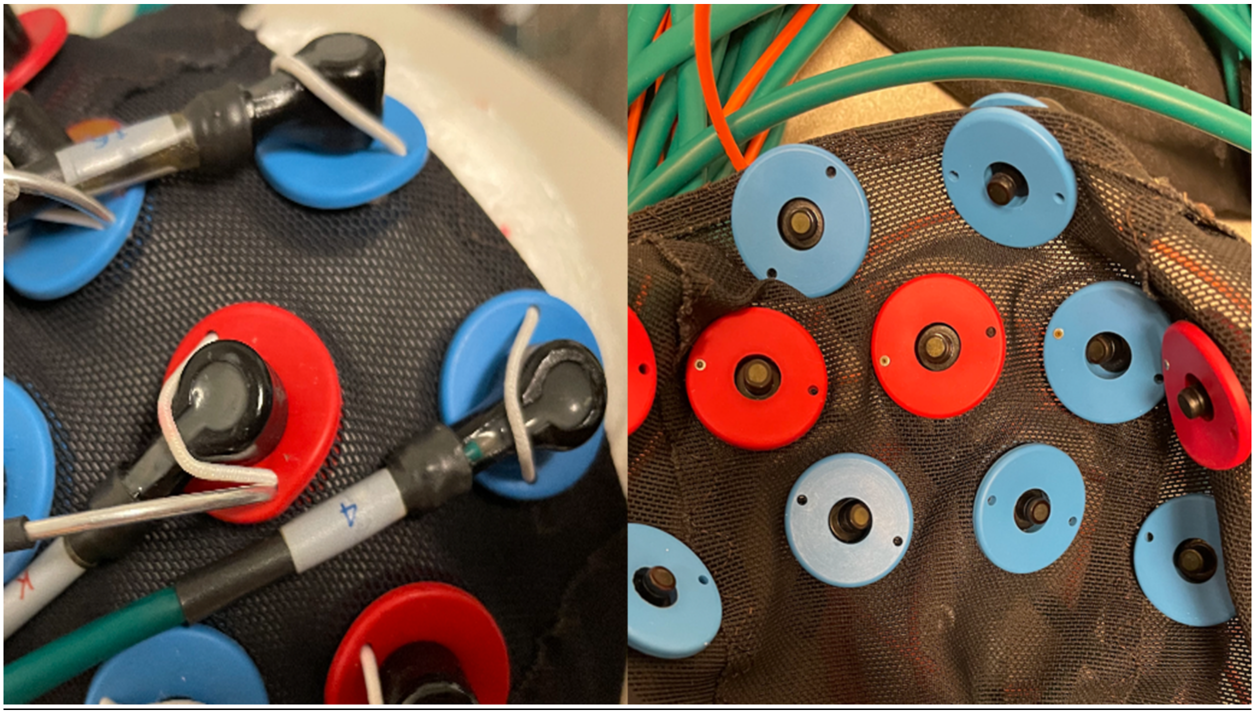

Fig. S1: Optode Placement. The fNIRS cap in **Fig. 2B** consists of nine sources held by red washers and fourteen adjacent short separation detectors held by blue washers.

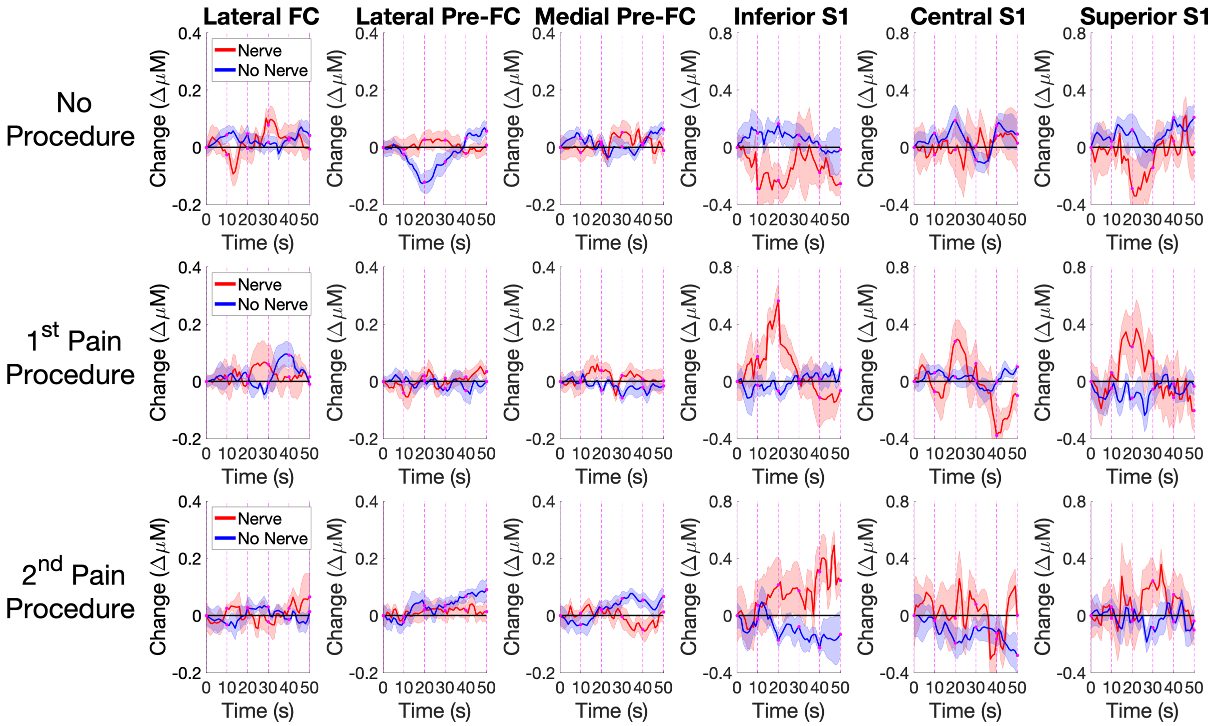

Fig. S2: Median Concentration Over Time. Observing the rolling median over a ten second window shows a similar trend to the mean concentration however there is no specific pattern that can be observed that only occurs during painful procedures.
